# DNA binds to a specific site of the adhesive blood-protein von Willebrand factor guided by electrostatic interactions

**DOI:** 10.1093/nar/gkaa466

**Published:** 2020-06-04

**Authors:** Angélica Sandoval-Pérez, Ricarda M L Berger, Adiran Garaizar, Stephen E Farr, Maria A Brehm, Gesa König, Stefan W Schneider, Rosana Collepardo-Guevara, Volker Huck, Joachim O Rädler, Camilo Aponte-Santamaría

**Affiliations:** Max Planck Tandem Group in Computational Biophysics, University of Los Andes, Cra. 1, 18A-12, 111711, Bogotá, Colombia; Faculty of Physics and Center for NanoScience, Ludwig-Maximilians-Universität München, Geschwister-Scholl-Platz 1, 80539 Munich, Germany; Maxwell Centre, Cavendish Laboratory, Department of Physics, University of Cambridge, J J Thomson Avenue, Cambridge CB3 0HE, UK; Maxwell Centre, Cavendish Laboratory, Department of Physics, University of Cambridge, J J Thomson Avenue, Cambridge CB3 0HE, UK; Department of Pediatric Hematology and Oncology, University Medical Center Hamburg-Eppendorf, Martinistr. 52, 20246 Hamburg, Germany; Department of Pediatric Hematology and Oncology, University Medical Center Hamburg-Eppendorf, Martinistr. 52, 20246 Hamburg, Germany; Department of Dermatology, Center for Internal Medicine, University Medical Center Hamburg-Eppendorf, Martinistr. 52, 20246 Hamburg, Germany; Maxwell Centre, Cavendish Laboratory, Department of Physics, University of Cambridge, J J Thomson Avenue, Cambridge CB3 0HE, UK; Department of Genetics, University of Cambridge, Cambridge CB2 3EH, UK; Department of Chemistry, University of Cambridge, Cambridge CB2 1EW, UK; Department of Dermatology, Center for Internal Medicine, University Medical Center Hamburg-Eppendorf, Martinistr. 52, 20246 Hamburg, Germany; Faculty of Physics and Center for NanoScience, Ludwig-Maximilians-Universität München, Geschwister-Scholl-Platz 1, 80539 Munich, Germany; Max Planck Tandem Group in Computational Biophysics, University of Los Andes, Cra. 1, 18A-12, 111711, Bogotá, Colombia; Interdisciplinary Center for Scientific Computing, Heidelberg University, Im Neuenheimer Feld 205, 69120 Heidelberg, Germany

## Abstract

Neutrophils release their intracellular content, DNA included, into the bloodstream to form neutrophil extracellular traps (NETs) that confine and kill circulating pathogens. The mechanosensitive adhesive blood protein, von Willebrand Factor (vWF), interacts with the extracellular DNA of NETs to potentially immobilize them during inflammatory and coagulatory conditions. Here, we elucidate the previously unknown molecular mechanism governing the DNA–vWF interaction by integrating atomistic, coarse-grained, and Brownian dynamics simulations, with thermophoresis, gel electrophoresis, fluorescence correlation spectroscopy (FCS), and microfluidic experiments. We demonstrate that, independently of its nucleotide sequence, double-stranded DNA binds to a specific helix of the vWF A1 domain, via three arginines. This interaction is attenuated by increasing the ionic strength. Our FCS and microfluidic measurements also highlight the key role shear-stress has in enabling this interaction. Our simulations attribute the previously-observed platelet-recruitment reduction and heparin-size modulation, upon establishment of DNA–vWF interactions, to indirect steric hindrance and partial overlap of the binding sites, respectively. Overall, we suggest electrostatics—guiding DNA to a specific protein binding site—as the main driving force defining DNA–vWF recognition. The molecular picture of a key shear-mediated DNA–protein interaction is provided here and it constitutes the basis for understanding NETs-mediated immune and hemostatic responses.

## INTRODUCTION

Release of DNA from neutrophils into the bloodstream to form neutrophil extracellular traps (NETs) is a key immune mechanism to trap and kill circulating pathogens (1,2). NETs are complex macro-molecular meshes, mainly composed of DNA, along with several scaffold proteins and highly-active antimicrobial agents. They efficiently ensnare and kill pathogens, triggered by diverse external stimuli. Since their discovery about 15 years ago, NETs have been attributed to provide a localized and timed immune response.

Misregulation of NETs is becoming increasingly linked to pathological conditions (3,4). In atherothrombosis, the chronic damage to endothelial cells up-regulates NETs, producing an arterial obstruction (5). In a plethora of autoimmune diseases, such as systemic lupus erythematosus, anti-neutrophil cytoplasmic antibodies-associated small vessel vasculitis, and rheumatoid arthritis, a limited clearance and up-regulation of NETs has been reported (6). Pregnancy complications and infertility have been linked to poor down-regulation of NETs (7). NETs are also found in patients with systemic infections (sepsis) (2,8,9) or in the respiratory tract of prone-to-infection cystic fibrosis patients (10). Presence of NETs within malignant tumors has been correlated with metastasis, indicating that NETs can promote cancer progression (11).

Once in the bloodstream, NETs must adhere to the blood vessels by establishing interactions with distinct blood proteins (12). However, the network of interactions stabilizing NETs remains largely unknown (3). An adhesive protein which is likely to play a key role in this process is von Willebrand Factor (vWF). vWF is an extracellular ultra-large protein which plays a vital role in primary hemostasis. Activated by the shear of flowing blood, vWF recruits platelets at sites of vascular injury, and thereby promotes the formation of plugs that stop bleeding (13,14). vWF is a multimeric protein composed of several dimers linked by disulfide bonds. Each dimer is formed by two identical monomers composed by several protein domains, which interact with various biomolecular partners. Mediated by flow-induced mechanical stress, vWF undergoes reversible conformational transitions from a globular to a stretched conformation, causing the exposure of cryptic binding sites, to thereby trigger vWF activation (15,16). These transitions occur at physiological shear-stresses typically found in venules and arteries of the order of 10 dyn/cm^2^ (17,18). Malfunction of vWF is related to several pathologies (19,20), ranging from acute bleeding to thrombotic disorders.

Of high relevance for many of these interactions is the vWF A1 domain. vWF anchors platelets (21), via the specific binding of the vWF A1 domain to the glycoprotein Ibα (GPIbα) receptor (22–24) in a shear-dependent manner (25–28). The A1 domain also interacts with the collagen matrix of sub-endothelial components (21). Furthermore, A1 has been shown to be auto-inhibited by interactions with its N-terminal linker, which connects A1 to the neighboring D’D3 domain (29), and with its C-terminal neighbor, the A2 domain (30–33). In addition, the interaction of A1 with the anticoagulant heparin (34,35), ristocetin (36,37), and with a single-stranded DNA fragment (ARC1172) (38) have been exploited in clinical applications.

vWF interacts with NETs (18,39) and, initially, the vWF–NETs interaction was suggested to be established via histones (40). Nevertheless, the recent work of Grässle *et al.* (18) revealed that vWF directly interacts with DNA from NETs, in a process of potential relevance during inflammatory and coagulatory conditions. The DNA–vWF interaction was found to be dependent on shear, to block the adhesion of platelets to vWF, and to be modulated by heparin. The authors suggested that a positively-charged region in the A1 domain may serve as the binding site for the negatively charged DNA molecule, and that DNA, platelets (via GPIbα), and heparin compete for this binding site. The possibility of direct vWF A1–nucleotide interactions is also supported by crystallographic studies of the ARC1172 single-stranded DNA fragment in complex with A1 (38). However, the molecular mechanism of interaction of double stranded DNA (ds DNA) from NETs with vWF remained to be elucidated. Here, we address this question by integrating atomistic molecular dynamics (MD), coarse-grained MD, and Brownian dynamics (BD) simulations, with microscale thermophoresis (MST), fluorescence correlation spectroscopy (FCS), gel electrophoresis, and microfluidic experiments. Our combined approach reveals a specific helical region in the vWF A1 domain, containing three arginine residues, as the main binding site for ds DNA. In turn, ds DNA seems to offer multiple unspecific binding sites for the binding of vWF A1, independently of the nucleotide sequence. The interaction between these two molecules has been found to be dominated by electrostatics. Our data explain the reduced binding of platelets in the presence of DNA by indirect steric clashes, rather than competition for the same specific binding site. Furthermore, the modulation imparted by heparin is attributed to partial overlap of the heparin and the ds DNA binding sites. All together, our study provides new molecular insights into the interaction of vWF with ds DNA, as basis to understand the formation, immobilization, and stability of NETs.

## MATERIALS AND METHODS

Equilibrium atomistic MD simulations were carried out, first, to monitor the spontaneous association of the vWF A1 domain to two different ds DNA fragments (PolyAT or ARC1172) and, second, to check the stability of predicted A1–ds DNA complexes after *in silico* mutations on the protein. Rigid body atomistic BD simulations were performed to dock vWF A domains to ds DNA and to estimate the association rate of A1 to the ds DNA ARC1172 fragment. Spontaneous association of vWF A1 to ds DNA was further explored using CG MD simulations.

Thermophoresis experiments probed the binding of isolated wild-type or mutated A1 domains to 23 base-pair ds DNA fragments and to ss DNA ARC1172 and examined the impact of ionic strength in the binding. FCS and electrophoretic mobility shift assays (EMSA) were used to characterize the interaction of different DNA fragments with vWF multimers and dimers, respectively, at varying ionic strengths, and under shear-stress conditions for the case of FCS. Microfludic experiments were conducted to monitor the formation of DNA–vWF conglomerates under shear-flow *ex vivo* conditions and to analyze their alterations due to a mutation on the A1 domain.

Simulation and experimental procedures are described in detail in the Supplementary text S1 (including Supplementary Table S1 and references (17,18,24,31,38,41–76).

## RESULTS

### Specific binding site in vWF A1 interacts with multiple unspecific sites in DNA

**Figure 1.**
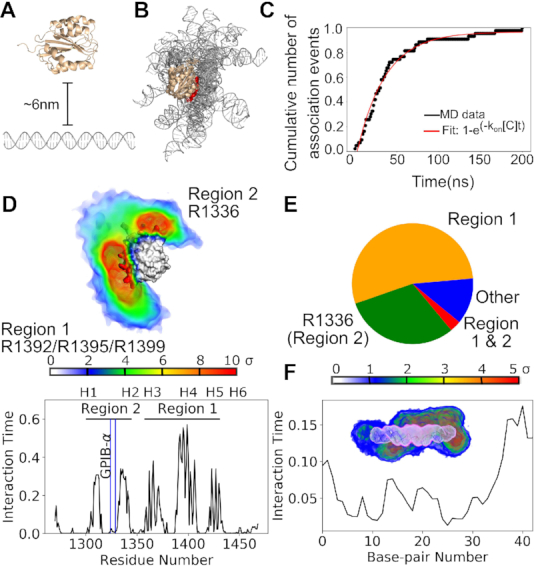
Spontaneous association of the vWF A1 domain and ds DNA probed by equilibrium atomistic MD simulations. (**A**) The vWF A1 domain (wheat) was initially separated by a distance of ≈6 nm from the ARC1172 ds DNA fragment (gray). Here, one out of the 42 different starting orientations is displayed. (**B**) 15 representative conformations of the vWF A1–DNA encounter complex are superposed, aligning the A1 domain to highlight the orientation variability of the bound ds DNA. Helix 4 (H4) of the A1 is highlighted in red. (**C**) Cumulative number of association events as a function of time at which the association took place (black) and a fit according to the indicated function (red). Number of association events is normalized with respect to the total number of simulations *N* = 42. (**D**) (Top) Time-average occupancy density of ARC1172 ds DNA around the A1 domain is presented (color-coded according to color bar: sigma units with 0 corresponding to the background average density). (Bottom) The interaction time of each residue of A1 with the ds DNA is displayed. Location of the helices (H1 to H6) along the sequence is indicated. Reported binding site for the platelet receptor GPIB-α (24) is highlighted by the blue line. The main binding region (region 1) corresponds to the arginines R1392, R1395, and R1399 at helix H4 (compare higher interaction time and larger density for that region with that for the rest of the protein). A second binding region (region 2) consist of arginine R1336 (at the loop connecting the beta strand 3 and helix H2). (**E**) Fraction of time the protein interacted with the ds DNA fragment is depicted: via R1392, 1395 and R1399 (Region 1: orange); R1336 (Region 2: green); both sets of arginines (regions 1 and 2: red), and other regions of the protein (blue). (**F**) Interaction time of each base-pair of the ds DNA ARC1172 fragment with the A1 domain. The inset shows the time-average occupancy protein density (blue to red) around the time-average occupancy DNA density (pink to white). The protein density is color-coded according to the shown color scale at different standard deviation (σ) units.

We first studied the spontaneous association of the vWF A1 domain to two different ds DNA fragments, by performing multiple unbiased atomistic MD simulations (84 runs, each one of 210 ns, for a total of 17.6 μs cumulative simulation time). Figure 1 shows the results for the ds DNA ARC1172 sequence (see results for the PolyAT sequence in Supplementary Figure S1). First, the protein was separated by approximately 6 nm from the ds DNA at different relative orientations (Figure 1A). In all the simulations, spontaneous binding events were observed (Figure 1B). Once the complex was established, the vWF A1 domain and the ds DNA fragments remained stably bound during the rest of the simulation (Supplementary Figure S2A). We monitored the moment in which A1 and ds DNA established the first contact (defined by the distance between these molecules getting smaller than 0.6 nm). 80% of these initial association events occurred within the first 50 ns of the simulations, ∼95% within the first 100 ns and the remaining 5% during the last 100 ns (Figure 1C). This indicates a very fast mode of association.

Multiple poses were identified for the vWF A1–DNA encounter complex (Figure 1B). Remarkably, despite this high conformational variability, the DNA targeted the same specific region in the vWF A1 domain (Figure 1D). This region consisted of the arginines R1392, R1395 and R1399, located at the helix 4 (H4) (Figure 1D). The dsDNA bound to this triad of arginines in the majority of the simulation time and to a minor extent to the arginine R1336 situated at the loop connecting the beta strand 3 and helix 2 (H2) (Figure 1D–E). Just in rare cases, a simultaneous interaction of dsDNA with the triad of arginines at H4 and R1336 was observed (Figure 1E). Reassuringly, the main binding region, largely overlaps with the binding site of the single-stranded ARC1172 DNA aptamer (38) (Supplementary Figure S2B).

Contrary to the specificity observed in the protein, multiple interaction sites on the DNA were observed for vWF A1 (Figure 1F). Although one of these sites presented higher interaction time than the others (namely, the end of the DNA fragment near base pair 40), we exclude this as a possible specific vWF binding site on the DNA as this interaction was not seen in neither the BD nor the CG simulations (see Figures 3 and 4 below).

A similar trend was observed for the spontaneous binding of the vWF A1 domain to the PolyAT sequence (Supplementary Figure S1). Hence, our extensive set of unbiased MD simulations indicates that vWF A1 contains a specific region for the binding of double-stranded DNA, which in turn offers multiple binding sites, indistinctly of the nucleotide sequence, for this protein domain.

**Figure 2.**
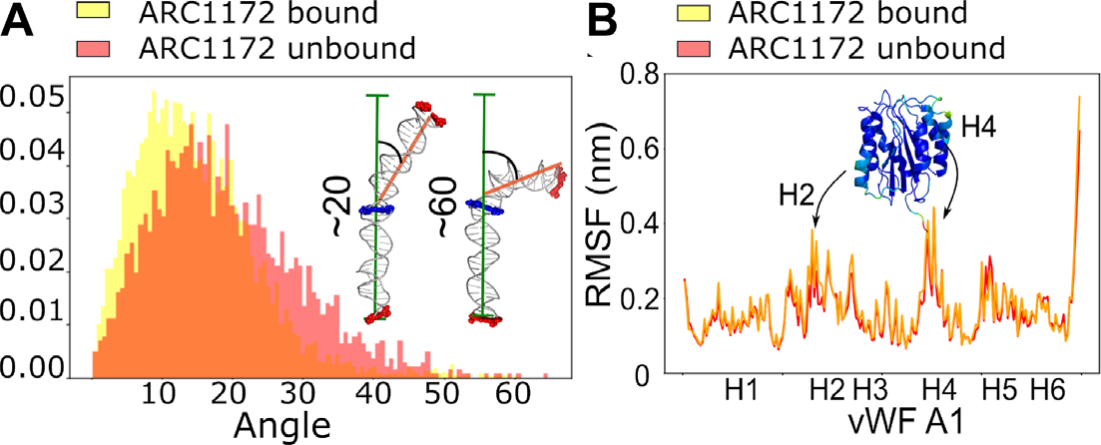
Internal dynamics of the ds DNA fragment and vWF A1 domain before and after association. (**A**) Bending angle distribution explored by ds DNA ARC1172 before (red) and after (yellow) binding to vWF A1. Both states, bound/unbound exhibited similar distribution as evaluated by the Kolmogorov-Smirnov test (*P*-value≥0.05 in 65% of 200 resample rounds, with N_bound_=60 and N_unbound_=23 randomly-chosen values for each round). Examples of bending angles acquired by the ds DNA fragment (≈20°: most frequent case , and ≈60°: a rarely explored highly bent conformation). (**B**) The root mean square fluctuation (RMSF) is presented along the A1 amino-acid sequence (helix positions indicated). The stability conferred by the Rossmann fold kept it invariable during the 17.6 μs of simulation time, independently of whether it was unbound (red) or bound (yellow) to the ds DNA.

Next, we investigated the changes in the internal dynamics of the vWF A1 domain and the ds DNA fragments upon binding. The ds DNA displayed different levels of bending, with a main bending angle of ∼20° (Figure 2A). The distribution of the bending angle, before and after ds DNA encountered the A1 domain, did not display statistically-significant differences (Figure 2A). In addition, local dynamics of ds DNA, monitored with properties such as internal rotations, internal translations, and average width and depth of major and minor grooves did not significantly vary upon binding of the DNA to the A1 domain (Supplementary Figures S3–S8). The A1 domain preserved its high rigidity, dictated by the Rossmann fold, after encountering the DNA fragment (Figure 2B). Note that these observations did not drastically vary by changing the nucleotide sequence (Supplementary Figure S9).

**Figure 3.**
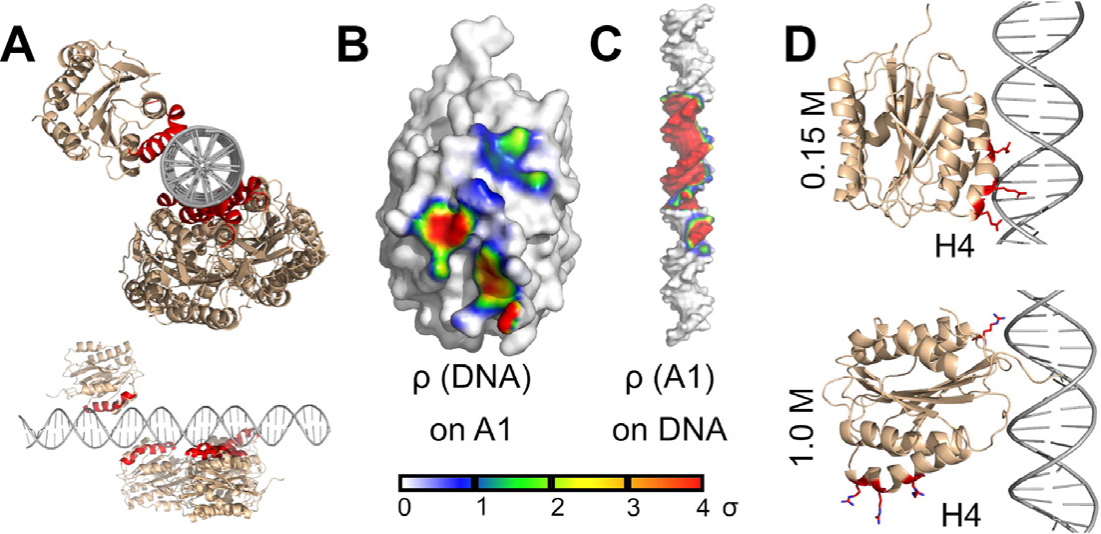
vWF A1–DNA interactions probed by rigid-body BD docking simulations. (**A**) Main docking conformations of the vWF A1 domain (wheat) around linear ARC1172 ds DNA (gray) are depicted, highlighting the helix H4 in A1 in red. Top and side views with respect to the ds DNA chain are shown. (**B**, **C**) Time-averaged occupancy density maps of ds DNA on A1 ρ(DNA) and vWF A1 on ds DNA ρ(A1), contoured at a surface of 0.6 nm away from the respective molecule. The density is displayed according to the shown color-scale at standard deviation (σ) units, after normalizing the map. Accordingly, the average density here has a value of zero. (**D**) Main poses of the vWF A1–ARC1172 ds DNA complex at 0.15 M (top) and 1.0 M (bottom) NaCl concentration, recovered from BD simulations differ (A1: wheat; DNA: gray and key arginines of A1: red).

To further validate the mode of interaction observed in MD simulations, we performed BD simulations. Here, the vWF A1 domain and the ds DNA were considered to be rigid bodies and the solvent was treated implicitly. These assumptions substantially enhanced the sampling. By conducting 20,000 rigid-body BD docking simulations for each ds DNA sequence, pre-selecting 500 structures considering those with lowest interaction energy, and clustering them, we obtained five representative poses of the vWF A1–DNA encounter complex (Figure 3A and Supplementary Figure S10). On the one hand, the time-averaged occupancy density map of ds DNA on the vWF A1 surface, retrieved by the BD simulations, revealed that ds DNA generally accommodates in front of helices H3, H4 and H5 (Figure 3B and Supplementary Figure S10). This region largely coincides with the main binding site identified in the MD simulations, i.e. the three arginines R1392, R1395, and R1399 (compare Figure 1D with Figure 3B). Other sites in the protein (such as R1336) were not captured by our BD-based docking selection, a result that is consistent with the low interaction time of DNA with other sides different than H4 observed in the MD simulations (Figure 1E). On the other hand, several interaction sites on the ds DNA fragment were observed for vWF A1 as confirmed by the existence of density at a region extending over one major groove and two minor grooves (Figure 3C). Note that here we preselected, energetically-favourable conformations, thus narrowing the contact region in the DNA. Similar results were found for the PolyAT ds DNA fragment, adding further evidence that the observed results are independent of the DNA sequence (Supplementary Figure S10). BD simulations also allowed comparison of the binding of ds DNA to the vWF A2 and A3 domains. This comparison highlights A1, among the three vWF A domains, as the main site responsible for the interaction with ds DNA, as previously suggested experimentally (18) (see supplementary text S2, Supplementary Table S2, and Figure S20).

**Figure 4.**
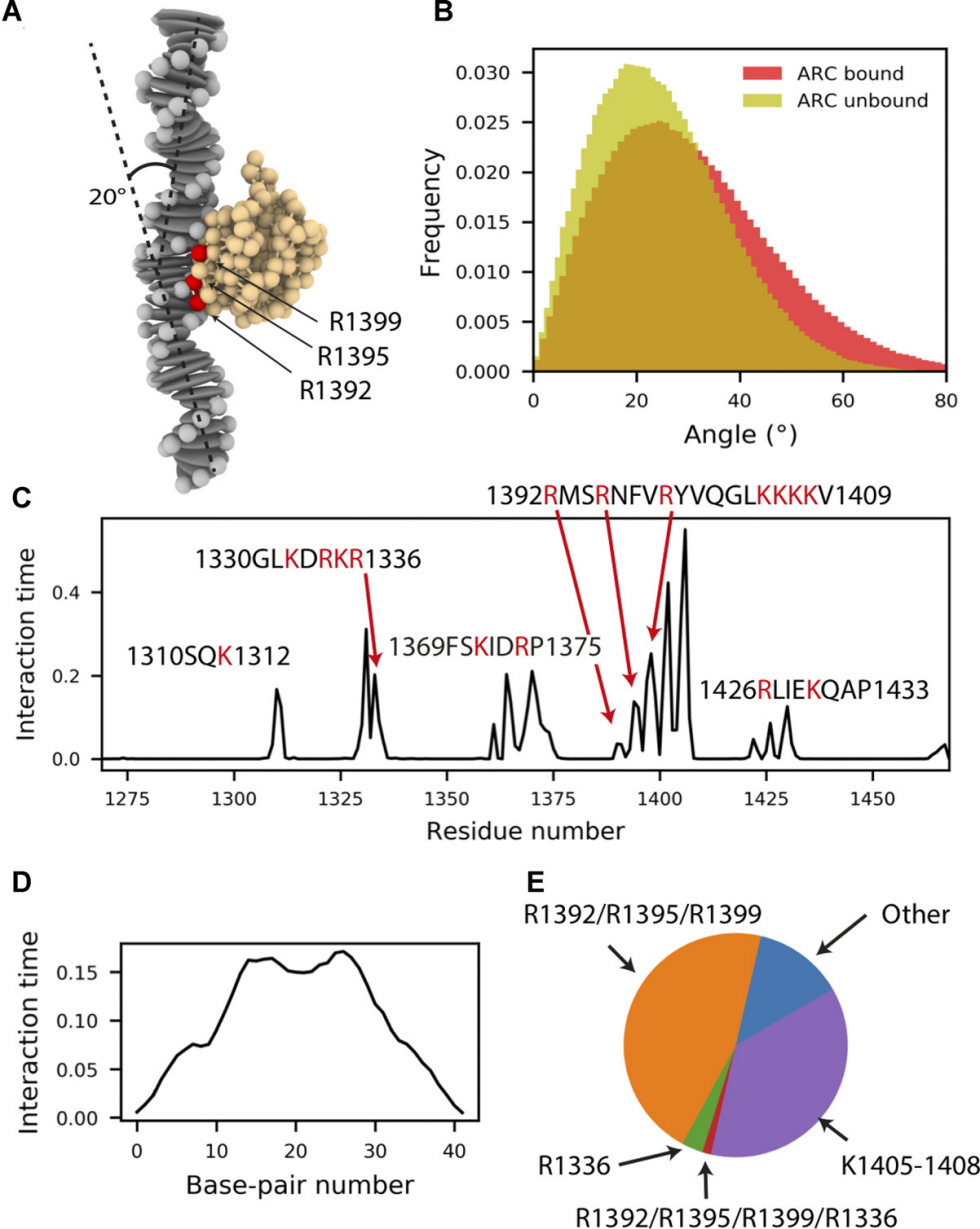
vWF A1–DNA interactions probed by CG MD simulations. (**A**) Representative conformation of the vWF A1–DNA encounter, picturing the main bending angle that the dsDNA fragment adopted. (**B**) The bending angle distribution in bound/unbound states exhibited similar distributions (*P*-value≥0.05 in 55% of 200 resample rounds, with N_bound_ =118 and N_unbound_=53 randomly-chosen values for each round). (**C**) Fraction of interaction time in which the residues of the vWF A1 domain are in in contact with the ARC1172 ds DNA fragment. (0: if they were never in contact, and 1: if they were in contact all the time). (**D**) Fraction of interaction time in which the base pairs are in contact with residues within the vWF A1 domain. (**E**) Fractions of interaction time that ds DNA spent with the the arginines triad R1392/R1395/R1399, the arginine R1336, both sets or arginines simultaneously, the Lysine patch (residues 1405–1408) or other regions of the protein.

CG MD simulations were also performed to assess mode of interaction between ds DNA and vWF A1, beyond atomistic time-scales, while enabling global flexibility of these two molecules (Figure 4). The ds DNA were coarse-grained using a modified rigid base-pair model, with an additional charged bead (*q* = −1 *e*) for each phosphate group. The protein was coarse-grained by mapping each amino acid in the crystal structure into a bead connected by an elastic network, conserving its secondary structure, and amino acids charge distribution (Figure 4A).

The dynamics of the unbound ds DNA fragment simulated in CG was consistent with that recovered by atomistic MD simulations (compare Figures 2A and 4B). Although, the bound distribution is slightly skewed toward larger bending angles in the CG simulations, possibly due to more thorough sampling, it also displays a small mean bending angle (∼20^*o*^) (Figure 4A–B). Furthermore, the bending-angle distribution for ds DNA in the bound and unbound states is statistically equivalent (*P*-value≥0.05 in 55% of 200 resample rounds, with Nbound =118 and Nunbound=53 randomly-chosen values for each round).

In agreement with the atomistic MD and BD simulations, ds DNA targeted the H4 on the A1 domain (Figures 4C, E), while sporadically bound to other regions of the protein. Interestingly, our CG simulations showed a higher interaction time of the DNA with the lysine patch K1405–1408, compared to the atomistic simulations (compare Figures 1D and 4C). This discrepancy emerges from the lysines and arginines in the CG model being described by spherical beads with identical charges and sizes, an approximation that limits the CG model from capturing subtle differences in the behaviour between these two residues. In addition, the level of coarse-graining of the protein (one bead per amino-acid) does not account for the higher exposure of the arginine side chains compared to that of the lysines in the helix H4 (≈40% higher solvent accessible surface was observed for R1392/R1395/R1999 than for K1405–1408 in the all-atom MD simulations). Nevertheless, when classifying the interaction time by the targeted region, it became clear that DNA interacted exclusively with R1392/R1395/R1999 in a major proportion than with the lysine patch K1405-1408 or with any other region of the protein (Figure 4E) Accordingly, the bent ds DNA allowed for a dual occupancy of the triad of arginines R1392/R1395/R1999 at H4 and the arginine R1336 only exceptionally (Figure 4E). The CG simulations also exhibited nonspecific interaction sites of the ds DNA (Figure 4D), particularly favorable at the middle of the fragment. Reassuringly, very similar results were obtained with the PolyAT sequence (Supplementary Figure S11). Thus, our CG simulations, taking into account DNA flexibility, further support our observations from atomistic MD and BD simulations.

### Electrostatics govern the vWFA1–DNA interaction

**Figure 5.**
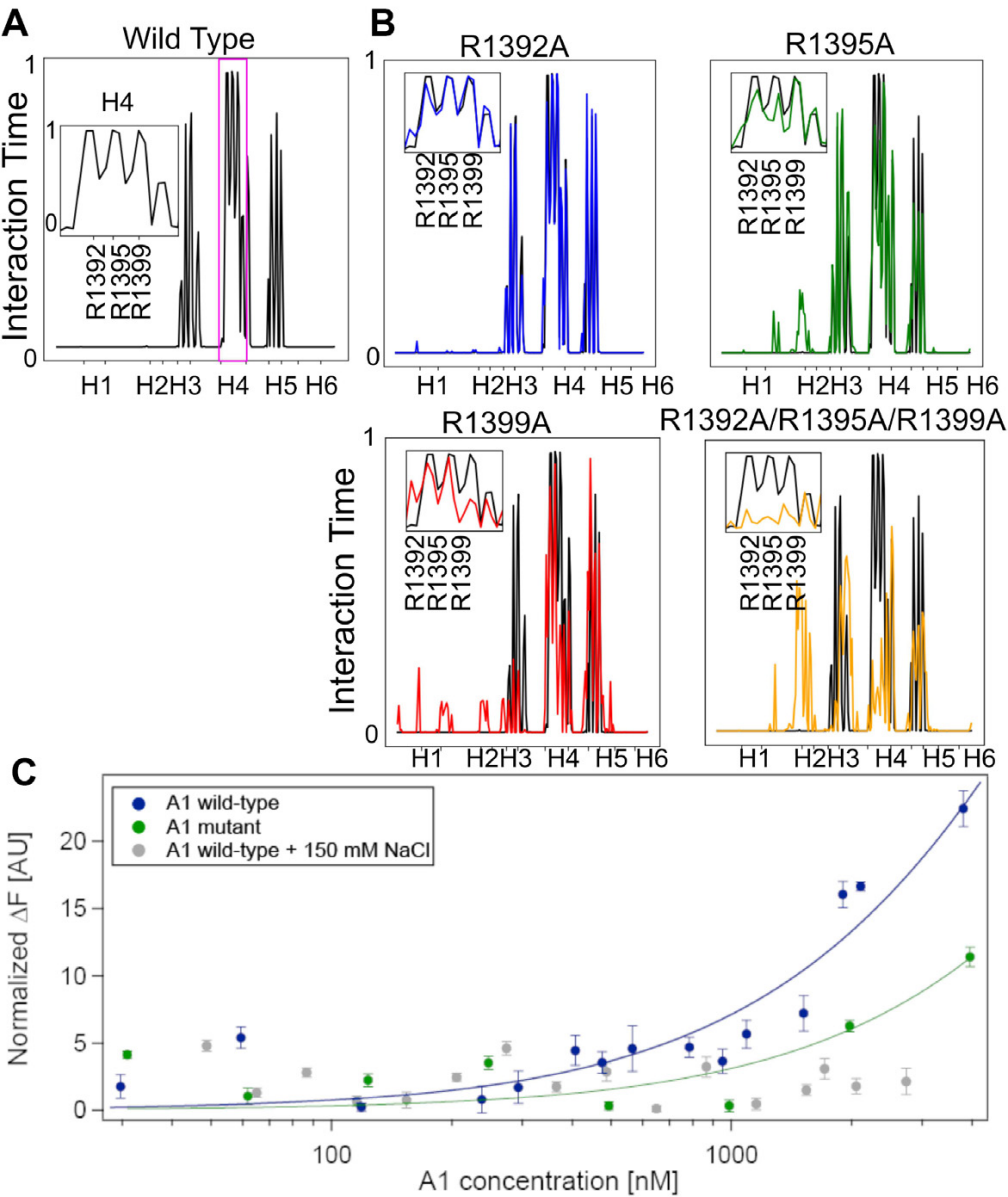
VWF A1–ds DNA interactions are governed by electrostatics. (**A**) Fraction of time in which the vWF A1 domain residues were in contact with the ARC1172 ds DNA fragment (0: they were never in contact; to 1: they were in contact all the time). The span of each helical secondary structure element of the A1 domain is highlighted in the X axis. The inset shows the interaction time with the three indicated arginines of helix 4. (**B**) Normalized interaction time between four representative vWF A1 mutants and ds DNA (color) is compared with the time for the wt protein (black), same format as in A. See results for all the studied mutants in Supplementary Figure S12. (**C**) Change in normalized fluorescence (Δ*F*) recovered from thermophoresis experiments was used to quantify binding of 23 base-pair ds DNA fragments to single vWF A1 domains. Titration curves correspond to the wt A1 domain in PBS buffer (blue) with fit *K*_*eq*_ = 13 ± 1 μM; the A1 domain with mutations R1392A, R1395A and R1399A (green) with fit *K_eq_* = 30 ± 6 μM, and the A1 domain in PBS with additional 150 mM NaCl (grey). Average ± standard error is shown in C (*N* = 4).

In the previous study of Grässle *et al.*, it was proposed that electrostatic interactions mediate the binding of vWF to ds DNA (18). A1 contains a highly positively-charged region, which could be complementary to the negatively-charged phosphate groups of ds DNA, located at its backbone but exposed to the solvent. Interestingly, in our atomistic, CG, and BD simulations, ds DNA was observed to mainly bind to a portion of this region, namely, the triad of arginines at H4 (Figures 1D–E, 3A, and 4C). This points to specific electrostatic interactions between these arginines and ds DNA as the main factor defining the vWF-DNA interaction. This hypothesis was tested by *in-silico* mutations (Figure 5A–B). The most energetically favorable conformation of the encounter-complex predicted by BD simulations was considered as the starting conformation for this test. Subsequently, the three arginines located in H4 were systematically replaced by either alanine or glutamic acid, resulting in 10 mutations. The mutated complexes were then simulated in multiple 210 ns MD runs (three replicas per mutant: cumulative time of 6.9 μs).

In accordance with our association simulations (Figures 1, 3 and 4), within the simulated time-scale, the wt A1 domain remained stably bound to the ds DNA fragment. Accordingly, R1392, R1395, and R1399 stayed more than 99% of the total simulation time in close contact with the ds DNA fragment (Figure 5A). The interaction-time of the vWF mutants with ds DNA was compared with that of the wild-type protein. We interpreted a reduction in the interaction-time to a destabilization of the vWF A1–ds DNA complex, imposed by the mutations (Figure 5B and Supplementary Figure S12). A broad level of destabilization was observed, ranging from single amino acid local changes (e.g. R1392A) to wider perturbations in which the complete protein-DNA interaction-profile changed (i.e R1395A, R1399A and R1392A/R1395A/R1399A). In the latter case, helix H4 partially (R1395A or R1399A) or completely (R1392A/R1395A/R1399A) lost contact with the ds DNA fragment, and thereby new interactions with other parts of the protein were established. For instance, this process occurred with helix H2 and its adjacent loops. The substitution of the arginine residues with glutamic acid (E) presented a similar, but stronger effect, to even display the extreme situation, for the triple mutant R1392E/R1395E/R1399E, in which the protein dissociated completely from the ds DNA fragment, in two out of three simulation replicas (Supplementary Figure S13). Remarkably, in all mutants in which R1399 was involved, there was a significant change of the protein-nucleotide interaction (Figure 5B and Supplementary Figure S12). Thus, this systematic *in-silico* mutation analysis demonstrates the relevance of the charged residues R1392, R1395, and R1399, particularly the latter, stabilizing the vWF A1–DNA interaction. The important role of these residues was further confirmed by mutations *in-vitro* (see sections below).

The influence of electrostatics on vWF A1–DNA interaction was further assessed by docking the vWF A1 domain to the ARC1172 ds DNA fragment, in rigid body BD simulations, varying the salt concentration from 0.05 M to 1 M (Figure 3D). When focusing on the predicted docking poses, it was observed that the protein reoriented, such that the protein-ds DNA contacts established via H4 at 0.15 M were changed by interactions with the residue R1336 at 1 M concentration. The dependency of the vWF A1–DNA encounter-complex conformation on the salt concentration observed here *in-silico* further stresses the role of electrostatics bringing these two macromolecules together.

### Association rate estimate

Our equilibrium simulations showed very fast vWF A1–DNA binding dynamics (Figure 1). Poor charge screening due to insufficient equilibration was discarded as the cause of this, because a converged number of ions around the DNA fragments was reached during the 10 ns of equilibration preceding the production runs (Supplementary Figure S14). A first-order-kinetics estimate of the association rate, derived by fitting a function 1 − exp(−*k*_*on*_[*C*]*t*) to the cumulative association-event curve (Figure 1C) and using a molecular concentration of [*C*] = 3.5 × 10^−5^ M (value determined by the given simulation box size), yielded an estimate of *k*_*on*_ of the order of 10^11^ M^−1^ s^−1^. This estimate is rather an upper boundary as it is based on the time the first contact was established and computed from 42 association events. An estimate based on the formation of several contacts and more association events was given by the BD simulations. At an ionic strength of 0.15 M, BD yielded *k*_*on*_ ∼ 10^9^ M^−1^ s^−1^, irrespective of the DNA nucleotide sequence. The two studied molecules are highly charged: the vWF A1 domain has a net charge *q*_*A*1_ = +6*e*, while the considered ARC1172 and PolyAT ds DNA fragments have a net charge of −82e. An estimate of the time τ, that such charges need to meet, assuming them to be point charges immersed in a bulk medium at 0.15 M ionic strength and using Debye-Hückel theory, was found to be τ = 34 ns (see supplementary text S3 and Supplementary Figure S21 for details of this calculation). This value is in very good agreement with the association time-scale observed in the MD simulations. Thus, strong long-range electrostatic attraction explains the fast first-contact encounter association kinetics observed here.

### Confirmation of the DNA binding site at A1 and the electrostatic nature of the vWF A1–DNA interaction *in-vitro* by thermophoresis experiments

To validate our computational predictions *in-vitro*, we performed MST binding measurements (Figure 5C). MST detects changes of the thermophoretic coefficient of the DNA upon binding. The technique has proven to be highly sensitive and allows the quantification of affinity constants ranging from pM to mM (77,78). We used recombinant A1 domain constructs, synthetic 23 bp ds DNA and ARC1172 ss DNA (see Materials and Methods). The normalized thermophoresis curves shown in Supplementary Figure S15 exhibit an upward shift for higher concentrations of A1 domains resulting from binding of the A1 domain to the DNA. Batch fitting of the change in normalized fluorescence as a function of protein concentration yields a dissociation constant for the 23 bp ds DNA of 13 ±  1 μM in PBS (see Figure 5C and Material and Methods for further details). Mutation of the A1 domain, by inserting neutral alanines instead of the positively charged arginines, leads to a weaker binding capacity of ds DNA (Figure 5C). Furthermore, increasing the ionic strength from 162 mM (PBS) to 212 mM (after addition of 150 mM NaCl) likewise decreased binding (Figure 5C). As a control, we also examined the binding of A1 to single-stranded ARC1172 DNA. For this case, the effect of the positively charged arginines has an even more pronounced effect (Supplementary Figure S15F). Here, the ss DNA binds to the unmodified A1 domain with a dissociation constant of 10 ±  2 μM, while for the mutant A1 a 10-fold higher value of 130 ± 7 μM was obtained. This supports the prediction that binding of single and double stranded DNA is dominantly driven by electrostatic interactions with the arginines at helix 4 of the A1 domain.

### FCS demonstrates that vWF multimers bind to ds DNA, independently of the DNA’s nucleotide sequence and length, and depending on the shear-stress and the ionic-strength

**Figure 6.**
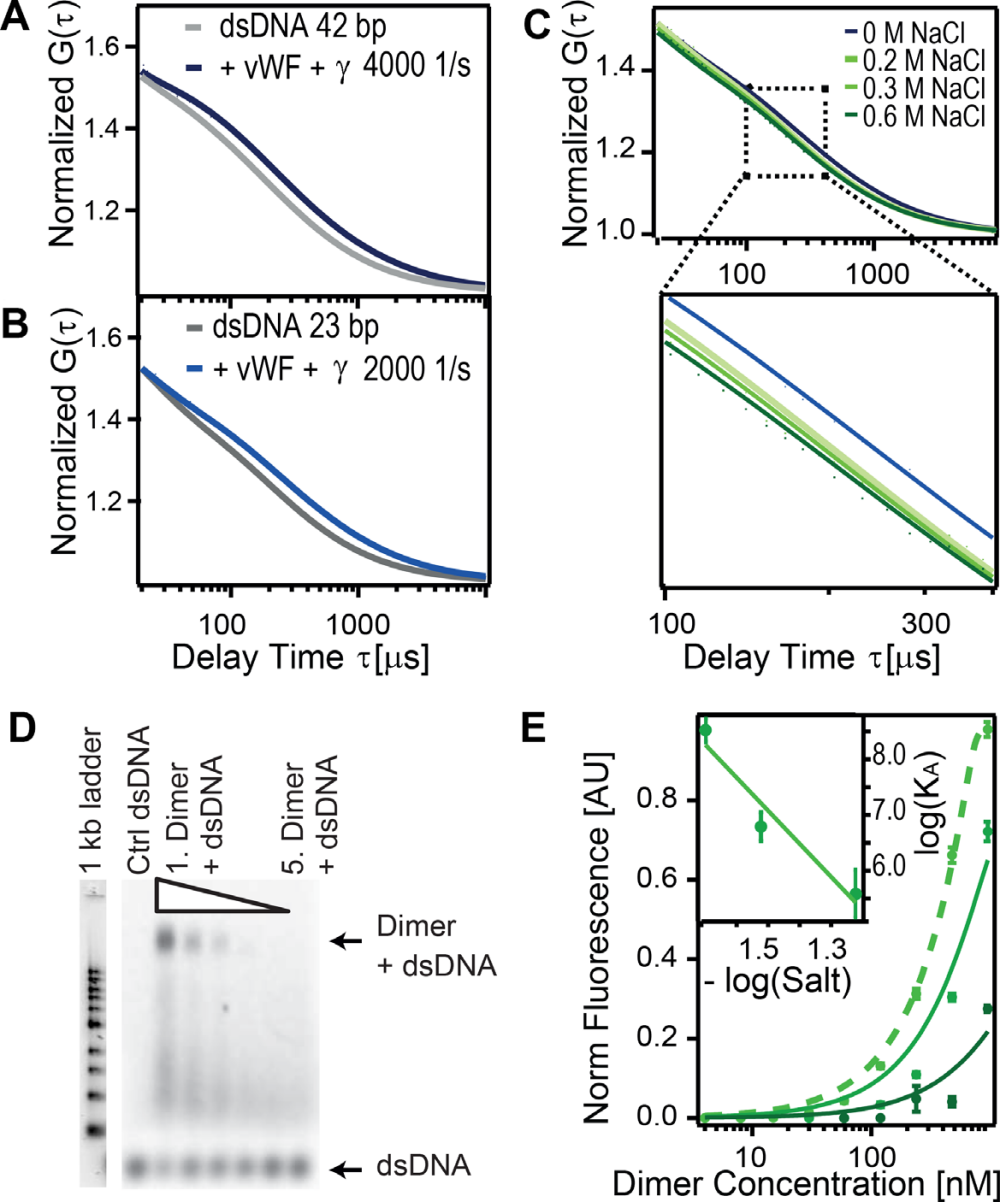
VWF multimers bind to ds DNA, independently of the nucleotide sequence and length of the DNA but sensitive to shear-stress and to the ionic-strength. (A, B) Normalized autocorrelation function G(τ) recovered from FCS is shown, for ARC1172 42bp (**A**) and for a 23bp (**B**) ds DNA alone (grey) and after vWF multimers were added and sheared (blue). The shear rate is indicated in the legend. (**C**) G(τ) for ARC1172 ds DNA at the indicated NaCl salt concentrations. (**D**) EMSA for ARC1172 42 bp ds DNA (*c* = 500 nM) binding to a concentration series of vWF dimer from 0.47 mg/ml to 15 μg/ml (20 mM ionic strength). Control (Ctrl) ds DNA contains 500 nM Cy5 labelled DNA only. See data for 30, and 60 mM in Supplementary Figure S19. (**E**) Normalized ds DNA fluorescence analyzed at the Dimer band for different ionic strengths of 20, 30, and 60 mM (light to dark green). Data was fitted with the general binding isotherm with *K*_*D*_ values from 21 ± 20 nM, 0.2 μM and 3 ± 1 μM, respectively. The inset shows the logarithm of salt concentration. The line depicts the fit log (*K*_*a*_) = *b* × log (*salt*) + *a*; with *b* = −6 ± 2 and *a* = −2 ± 2.

Next, we expanded our study, beyond single vWF A1 domains, by focusing on the more physiologically-relevant situation of vWF multimers. We monitored the association of ds DNA to either full-length vWF multimers or vWF dimers, using FCS. Because the binding site in the A1 domain is hidden in full-length vWF and only becomes accessible in the stretched conformation of vWF, we used a shear cell combined with fluorescence correlation spectroscopy (FCS) as we have introduced earlier (74,75). In short, the set-up allows the application of a constant shear flow in a Couette-type flow chamber and immediately afterwards, the performance of FCS measurements. We used fluorescently labeled ds DNA and measured the diffusion constant via FCS. Binding of two molecules results in an increased hydrodynamic size, thereby shifting the autocorrelation function towards larger diffusion times. This was effectively the case for the two tested ds DNA sequences, when vWF was added and the sample was sheared with a shear rate of γ = 2000– 4000 s^−1^ (Figures 6A–B). For the 23 bp DNA, a two-component fit of the autocorrelation curves yields a fraction of 21 ± 2 % in the bound state. Supplementary Figure S16 shows that the controls, ds DNA without vWF and shearing and ds DNA with vWF but no shearing, did not show a binding-induced shift in the autocorrelation curve. This indicates that opening the vWF stem to expose the A1 domain is crucial for ds DNA binding. In the presence of vWF, a higher shear rate of γ =  4000 s^−1^ has no substantial effect on the fraction of ds DNA bound to wild-type vWF ($23 \pm 2\, \%$). Increasing the ionic strength of the buffer by adding NaCl reduces binding as shown in Figure 6C. The addition of 150 mM NaCl decreases the fraction of bound DNA by 10%. Taken together, the FCS results confirm that vWF binds ds DNA independently of the DNA sequence and requires shear force to expose the A1 domain. As expected for an electrostatic interaction, the fraction of bound ds DNA decreases with increasing ionic strength.

To further validate these results, electrophoretic mobility shift assays (EMSA) were performed. Here, the binding of ARC1172 ss DNA and ds DNA (42 bp), as well as the 23 bp DNA, to eGFP full-length dimer or wt vWF were studied (Figure 6D and Supplementary Figures S17–S19). For the 23 bp DNA, the binding was too weak to be determined via EMSA (Supplementary Figure S17). However, for the ARC1172 ss DNA a clear band shift of vWF dimer and a dominant fluorescence signal of the Cy5 labelled DNA at the dimer band could be extracted (Supplementary Figure S18). Evaluating the fluorescence intensity at the dimer band for the different concentrations of ds DNA allows the extraction of a *K_D_* = 1 ± 6 nM (Supplementary Figure S18C). These results suggest a strong binding of ss DNA to vWF in the nM range which is in accordance to literature (38). Also, for the ARC1172 ds DNA, binding to the full-length vWF dimer was observed at ionic strength from 20 mM to 60 mM (Figure 6D and Supplementary Figure S19). We found that small changes in the ionic strength of the buffer from 20 mM to 60 mM drastically reduced the binding strength by 4 orders of magnitude, from 3 nM to 3 μM (Figure 6E). A linear regression to the log-log curve of the association constant as a function of the salt concentration yielded log(*K*_*A*_) = (−6 ± 2) × log(salt) − (2 ± 2) (Figure 6E, inset). According to the counter-ion condensation concept (79–81), the slope relates to the number of counter-ions released by DNA upon binding to the protein, while the intercept relates to the non-electrostatic (or salt-independent) component of the interaction. The obtained slope of –6 ± 2 is consistent with the number of DNA-binding arginines observed in the simulations (three arginines R1392, 1395 and R1399 per A1 domain times 2 A1 domains per vWF dimer). Extrapolated to a concentration of the order of ≈100 mM, the salt-dependent component, (−6 ± 2) × log(0.1) = 6 ± 2, is about three-fold the salt-independent part (namely, the intercept of −2 ± 2 ). Interestingly, when extracting the DNA-A1 potential interaction energy from the MD simulations and splitting this quantity into electrostatic (Coulomb) and short-range (Lennard–Jones) contributions, we obtained a similar three-fold proportion: −441 ± 56 kJ/mol for the Coulomb and −129 ± 20 kJ/mol for the Lennard–Jones parts (average ± stdev). This analysis suggests that the salt-dependent component dominates at this range. Nevertheless, overestimation of the binding strength by EMSA as well as large susceptibility of vWF to environmental electrostatic changes, i.e. calcium, salt and pH (29,73,82–84) may also add to this interpretation.

### Demonstration of the DNA binding site in the vWF A1 domain by microfluidic experiments

FCS and EMSA results confirmed that the shear-dependent binding of full-length wt vWF to ds DNA is sensitive to the surrounding ionic strength while independent from the ds DNA nucleotide sequence. We finally examined whether this interaction can be significantly diminished, under shear flow conditions, by the replacement of R1399 by alanine, as our simulations and thermophoresis measurements predicted it should be the case. Accordingly, functional microfluidic experiments were performed in wt vWF- or R1399A vWF-coated channels, perfused with a solution containing ds DNA and wt vWF or the above-mentioned vWF mutant. Floating conglomerates of ds DNA in the presence of wt vWF in the perfusion medium could hardly be recognized in the still images. Nevertheless, they could be detected when aggregate motion tracking of images was implemented. This visualization approach, consisting of the superposition of several images of different times, has been proven successful in visualizing rolling vWF-based conglomerates (31,85).

**Figure 7.**
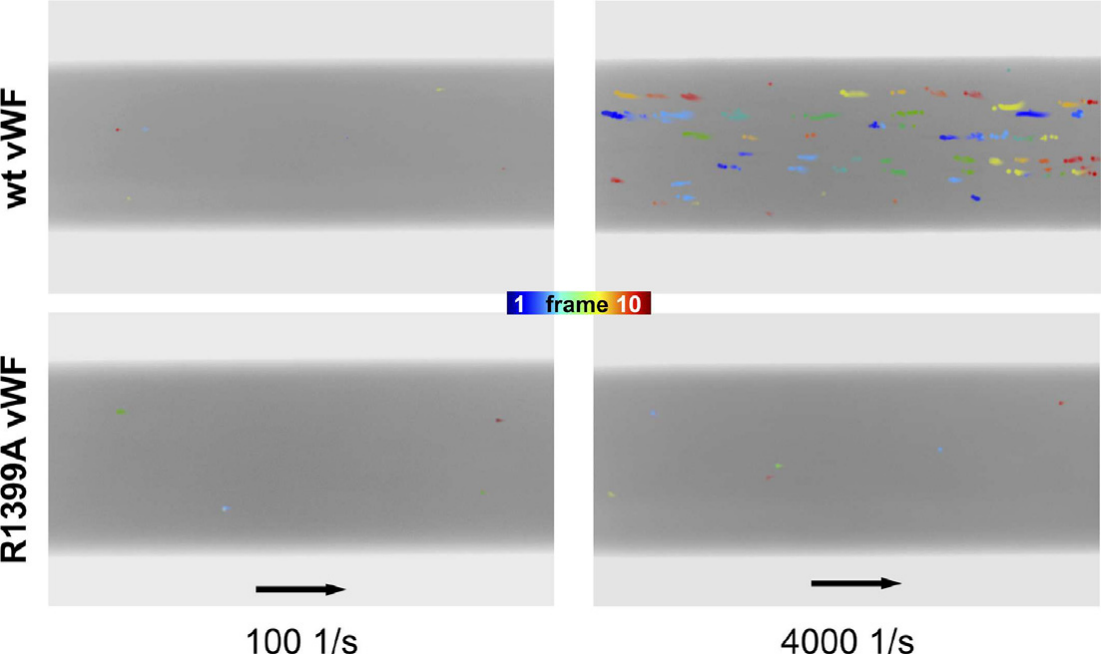
Binding of full-length vWF to ds DNA under shear flow conditions was captured by microfluidic experiments. Motion tracking of aggregates composed of ds DNA and wildtype vWF (upper row) and to vWF with the mutation R1399A in the A1 domain (lower row) is presented. Image compositions of 10 sequential frames each, taken at a frequency of 1.25 images per second, are shown highlighting the floating conglomerates in color. 60 s of high shear application of 4000 s^−1^ resulted in wt vWF-DNA conglomerates (upper right). In contrast, vWF–DNA interactions are not detectable neither for the vWF mutant R1399A nor for the case of the low shear stress of 100 s^−1^. Flow direction is indicated with black arrows corresponding to 100 μm.

After the application of a low shear of 100 s^−1^ for 60 s, we could only detect a diffuse unstructured background fluorescence emission, for both experimental groups, which could be attributed to floating pre-stained ds DNA (Figure 7, left). Pretreatment with a physiological shear rate of 4000 s^−1^ (which is a value large enough to stretch free-floating VWF (18) for 60 s resulted in floating conglomerates of ds DNA in the presence of wt vWF in the perfusion medium, detectable on a focal plane near the bottom of the channels (Figure 7, right). In the same shear regime, no conglomerates emerged in the presence of R1399A vWF instead of wt vWF (compare top and bottom panels of Figure 7 and see supplementary Movie S1) as well as in the complete absence of vWF in the perfusion media (data not shown). Taken together, our microfluidic findings confirm the assumption of ds DNA binding to the A1 domain to thereby form shear-sensitive VWF–DNA conglomerates. Furthermore, they prove the essential role of R1399 in A1 for the ds DNA–vWF interaction.

## DISCUSSION

Here, we have characterized in detail the interaction of vWF with ds DNA fragments by using a combination of atomistic and coarse-grained simulations and biophysical experiments. Extensive simulations, combining atomistic MD (Figure 1 and Supplementary Figure S1), atomistic BD (Figure 3 and Supplementary Figure S10), and CG MD (Figure 4 and Supplementary Figure S11) association simulations and a systematic *in-silico* stability mutation scan (Figure 5B and Supplementary Figure S12), demonstrate that a specific region in the vWF A1 domain, namely helix 4, specifically interacts with ds DNA. The arginines R1392, R1395 and R1399 were found to be the key residues participating in this interaction. These observations were further validated by thermophoresis experiments, in which the wt vWF A1 domain showed higher binding to ds DNA fragment than the mutant R1392A/R1395A/R1399A did (Figure 5C and Supplementary Figure S15F). Additionally, microfluidic experiments confirmed the decisive role of the helix 4 in the protein for binding to ds DNA, in the relevant case of vWF multimers, *in-vitro*, under shear-stress conditions (Figure 7 and Movie S1). The determined binding region is also consistent with the binding site for the single-stranded ARC1172 DNA aptamer (38).

Note that the distant R1336 also bind to ds DNA (Figure 1D). However, the interaction time of ds DNA with the region containing this residue was smaller than that with the triad of arginines at Helix 4 (Figures 1D–E and 4C,E). Furthermore, the contacts this residue established with ds DNA augmented upon destabilization of the main interaction site at helix H4 either by *in-silico* mutations (Figures 5A–B) or by increasing the ionic strength in the BD simulations (Figure 3D). Thus, we think R1336 contributes only weakly to the interaction with ds DNA, while the triad of arginines at helix 4 constitutes the main binding site to DNA. In consequence, our work highlights the central role of the vWF A1 as a hub in blood, serving as a binding site, not only for GPIB-α-platelet complexes and collagen during primary hemostasis (13), but also for molecules used for clinical proposes such as heparin (86) and ARC1772 single-stranded DNA (38), and remarkably for ds DNA during the formation of NETs.

Our simulations revealed a small amount of bending of DNA and this motion occurred independently of whether DNA was bound or not to the very rigid A1 domain (Figures 2 and 4). Furthermore, protein–DNA contacts occurred more predominantly with the phosphate groups than with the base pairs (Figure 1F and 3C). Moreover, our simulations combined with FCS showed the ability of vWF to bind to ds DNA independently of the DNA sequence (Figures 1, 3, 4, 6 and Supplementary Figures S1, S10–S11). These findings are consistent with conformational selection, irrespective of the DNA nucleotide sequence, as the main mechanism defining the recognition of vWF-A1 by ds DNA, further evidencing this as the dominant situation for protein–DNA binding (87).

Association of vWF A1 to the DNA fragments was an extremely fast process, with rates for the formation of the first contact of the order of 10^11^ M^−1^s^−1^ in MD simulations and going down to rates ∼10^9^ M^−1^s^−1^ for the formation of the most stable complex during BD simulations. The estimates from BD are about one order of magnitude higher than the value for highly-charged fast protein-protein complex binders, such as the barnase-barstar complex (2.8 × 10^8^ M^−1^ s^−1^) (88), but are comparable with the reported value for protein–DNA complexes (in the range of 10^9^ M^−1^ s^−1^) (89). Given the large charge of the vWF A1 domain and the ds DNA fragments, the encounter time at a typical physiological concentration from Debye Hückel theory was estimated to be in the order of tens of nanoseconds. In consequence, strong long-range electrostatic attraction appears to be a key factor governing the fast association kinetics observed here. The estimation of the dissociation rates from the simulations was not practical. However, thermophoresis experiments enabled the determination of equilibrium constants. They were in the order of 10 micromolar for both ds DNA and ss DNA ARC1172 fragments binding to A1 (Figure 5C and Supplementary Figure S15). Note that the previous estimates for ss DNA ARC1172 were in the nanomolar regime (38). We recover those when using vWF dimers instead of single A1 domains, in electrophoretic mobility shift assays (Figure 6D–E), and attribute the increment in binding affinity to the existence of two A1 domains in the dimer. The binding strength in these assays also displayed a large sensitivity to changes in ionic strength (Figure 6E and Supplementary Figure S19). In the light of the counterion condensation concept (79–81), the electrostatic (salt-dependent) component of the interaction was found to be consistent with two triads R1392/1395/R1399 per vWF dimer displacing six ions upon binding of vWF to DNA, while the non-electrostatic (salt-independent) contribution was estimated to be about one third of the salt-dependent part at a ionic concentration ≈100 mM. The potential energy derived from the simulations, interestingly, showed a similar proportion of electrostatic versus short-range interactions. Thus, these data suggest a dominance of salt-dependent over salt-independent contributions. However, EMSA might overestimate the reduction in binding strength, as it does not allow for characterization in shear-activated conditions. Moreover, vWF is an enormous multi-domain protein that is very susceptible to environmental electrostatic changes (see below). Accordingly, we can not exclude the ionic strength to also affect conformational features of the protein in addition to its influence on the release of counterions bound to the DNA.

Electrostatic forces play a key role in vWF. Calcium is important for the function of several of its domains (82); the dimer conformation is tightly modulated by pH changes in a process involving the D’D4 domain (73,83), and recently the binding of platelets to vWF was observed to be highly modulated by charge residues (29) and electrostatic steering (84). Our combined data from simulations, MST, and FCS provide further insights, by demonstrating how the interaction of vWF with ds DNA is also electrostatically driven, with three positively charged residues decisively mediating the interaction.

**Figure 8.**
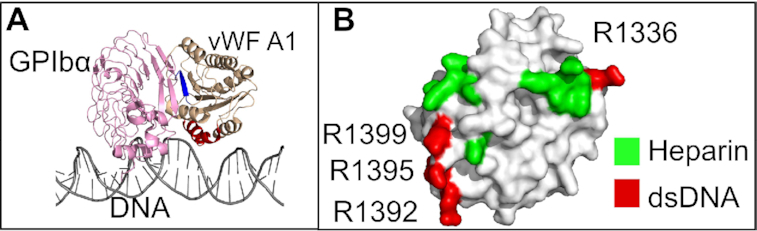
Comparison of binding sites in the vWF A1 domain. (**A**) The X-ray structure of the vWFA1–GPIb complex (PDB id.1SQ0 (24)) and that of the here-predicted vWF A1–DNA fragment complex (corresponding to the best-ranked pose recovered from BD docking simulations) are overlapped (vWF A1: wheat; DNA: gray and GPIBα: pink). Binding sites for GPIBα and DNA are shown in green and red, respectively. The linear conformation of DNA presented here is consistent with the small bending angles sampled by the DNA when interacting with the A1 (Figure 2A). (**B**) Residues related to heparin binding (34,35) are mapped on the A1 surface (green) and contrasted to the main three arginines identified here to bind to ds DNA (red).

Coming back to the formation of NETs, in previous experimental studies, the binding of extracellular DNA from NETs to vWF was observed to be triggered by the shear of the flowing blood (18). Our FCS (Figures 6A–C) and microfluidic experiments (Figure 7 and Movie S1) recapitulated the key role shear-stress has in triggering the ds DNA–vWF interaction. In addition, the presence of DNA from NETs was previously shown to block the binding of platelets to vWF and, furthermore, the inclusion of heparin reduced the binding of DNA to vWF (18). Thus, it was suggested that ds DNA, heparin, and GPIb-α compete for the same binding site on the vWF A1 domain (18). Our calculations provide a molecular framework for these observations. When overlapping the structure of the vWF A1-GPIb-α complex with that predicted here for the complex vWF A1–DNA fragment, we observe that binding sites, although close to each other, are different (beta-strand 3 for GPIb-α and helix 4 for DNA in Figure 8A). Our study thus suggests that the experimentally-observed blockage of platelet–vWF binding, due to the presence of DNA from NETs, is not caused by competition for the same specific binding site at vWF, but rather by indirect steric clashes of these two macromolecules when associating to A1. In addition, when mapping the residues involved in heparin-binding (90) together with those identified here for ds DNA onto the surface of A1, it becomes clear that DNA would not only cause indirect steric occlusion of heparin-binding residues, but also would directly compete for R1395 (Figure 8B) and this may be the cause of the heparin-size dependency for vWF–DNA binding (18). Interestingly, the mutation R1399H has been reported to cause a defect in collagen binding (91,92). This mutation affects one of the arginines identified here for ds DNA binding. Accordingly, we predict that binding of collagen to A1 may also be hindered by the interaction of ds DNA to this domain. Nevertheless, the ds DNA–vWF interaction mechanism is still compatible with the overall binding of vWF to collagen, as its major binding site is located in the A3 domain.

## CONCLUSIONS

The blood protein vWF was shown recently to interact with extracellular DNA from NETs (18). Here, we provide an explanation of the molecular interplay between these two molecules, by a combined approach using MD, BD and CG simulations, together with thermophoresis, FCS, EMSA, and microfluidics. We demonstrate that a specific helix of the vWF A1 domain is responsible for the interaction of vWF with ds DNA and that electrostatics play a key role in defining this interaction. The ds DNA displayed minor conformational changes upon binding to vWF and the binding was rather independent of the DNA sequence. Furthermore, we confirmed shear-stress to be essential to trigger the binding of ds DNA to vWF multimers. All this together, points to conformational selection —guided by electrostatics—as the key factor driving vWF–ds DNA recognition, once the A1 becomes exposed due to the action of shear. Our data attributes the observed platelet-binding reduction upon DNA binding to indirect steric clashes rather than direct competition for the same binding site. Furthermore, the observed heparin-size impact on the vWF–DNA interaction is explained by a partial overlap of the binding sites of heparin and DNA. It is tempting to speculate that shear-mediated binding of extracellular DNA to a specific region in the vWF A1 domain, near the platelet binding site, confers stability to the NETs while balancing their immune and coagulation responses in the blood. It will be highly interesting to investigate this hypothesis in future studies.

## Supplementary Material

gkaa466_Supplemental_Files
