# DNA binds to a specific site of the adhesive blood-protein von Willebrand factor guided by electrostatic interactions

**DOI:** 10.1093/nar/gkaa988

**Published:** 2020-10-22

**Authors:** Angélica Sandoval-Pérez, Ricarda M L Berger, Adiran Garaizar, Stephen E Farr, Maria A Brehm, Gesa König, Stefan W Schneider, Rosana Collepardo-Guevara, Volker Huck, Joachim O Rädler, Camilo Aponte-Santamaría

**Affiliations:** Max Planck Tandem Group in Computational Biophysics, University of Los Andes, Cra. 1, 18A-12, 111711, Bogotá, Colombia; Faculty of Physics and Center for NanoScience, Ludwig-Maximilians-Universität München, Geschwister-Scholl-Platz 1, 80539 Munich, Germany; Maxwell Centre, Cavendish Laboratory, Department of Physics, University of Cambridge, J J Thomson Avenue, Cambridge CB3 0HE, UK; Maxwell Centre, Cavendish Laboratory, Department of Physics, University of Cambridge, J J Thomson Avenue, Cambridge CB3 0HE, UK; Department of Pediatric Hematology and Oncology, University Medical Center Hamburg-Eppendorf, Martinistr. 52, 20246 Hamburg, Germany; Department of Pediatric Hematology and Oncology, University Medical Center Hamburg-Eppendorf, Martinistr. 52, 20246 Hamburg, Germany; Department of Dermatology, Center for Internal Medicine, University Medical Center Hamburg-Eppendorf, Martinistr. 52, 20246 Hamburg, Germany; Maxwell Centre, Cavendish Laboratory, Department of Physics, University of Cambridge, J J Thomson Avenue, Cambridge CB3 0HE, UK; Department of Genetics, University of Cambridge, Cambridge CB2 3EH, UK; Department of Chemistry, University of Cambridge, Cambridge CB2 1EW, UK; Department of Dermatology, Center for Internal Medicine, University Medical Center Hamburg-Eppendorf, Martinistr. 52, 20246 Hamburg, Germany; Faculty of Physics and Center for NanoScience, Ludwig-Maximilians-Universität München, Geschwister-Scholl-Platz 1, 80539 Munich, Germany; Max Planck Tandem Group in Computational Biophysics, University of Los Andes, Cra. 1, 18A-12, 111711, Bogotá, Colombia; Interdisciplinary Center for Scientific Computing, Heidelberg University, Im Neuenheimer Feld 205, 69120 Heidelberg, Germany

*Nucleic Acids Research*, 2020, 48(13): 7333–7344, https://doi.org/10.1093/nar/gkaa466

The authors would like to apologize for mistakes in the *P*-value calculations of the DNA bending angle distributions. The following corrections have been made to the published article.

**SUPPLEMENTARY TEXT**

**S1 SUPPORTING MATERIALS AND METHODS**

**Atomistic Molecular Dynamics (MD) simulations**

*Analysis of atomistic MD simulations*

The distributions of the bending angle before and after association were compared using the two-sample Kolmogorov-Smirnov test (22).

has been corrected to

The distributions of the bending angle before and after association were compared using the two-sample Kolmogorov-Smirnov test (22). The test was carried out for a subset of N_bound_ and N_unbound_ uncorrelated angle values which were randomly-chosen from the angle time traces, in the bound and unbound states, respectively. To avoid correlation between chosen data points, the values were separated by a time window of at least 10 to 100 ns (time in which the autocorrelation function of the angle traces diminished from 1 to 0.1), thus yielding sample sizes between 23 and 152. The test was repeated 200 times considering different N_bound_ and N_unbound_ values and the number of cases the *P*-value was larger than 0.05 was quantified.

**Figure 2A**

Both states, bound/unbound exhibited similar distribution as evaluated by the Kolmogorov-Smirnov test (D = 0.13 and *P*-value = 0.34).

has been corrected to

Both states, bound/unbound exhibited similar distribution as evaluated by the Kolmogorov-Smirnov test (*P*-value≥0.05 in 65% of 200 resample rounds, with N_bound_=60 and N_unbound_=23 randomly-chosen values for each round).

**Figure 4B**

The bending angle distribution in bound/unbound states exhibited similar distributions (Kolmogorov–Smirnov test: D = 0.18 and *P* = 0.08).

Has been corrected to

The bending angle distribution in bound/unbound states exhibited similar distributions (*P*-value≥0.05 in 55% of 200 resample rounds, with N_bound_ =118 and N_unbound_=53 randomly-chosen values for each round).

**RESULTS**

**Specific binding site in vWF A1 interacts with multiple unspecific sites in DNA:**

Furthermore, the bending-angle distribution for ds DNA in the bound and unbound states is statistically equivalent (Kolmogorov–Smirnov test: D = 0.18, *P* = 0.08).

Has been corrected to

Furthermore, the bending-angle distribution for ds DNA in the bound and unbound states is statistically equivalent (*P*-value≥0.05 in 55% of 200 resample rounds, with Nbound =118 and Nunbound=53 randomly-chosen values for each round).

**Figure S9**

Both states, bound and unbound, exhibited similar distribution as evaluated by the Kolmorov-Smirnov test (D=0.11 and *P*-value=0.58).

Has been corrected to

Both states, bound and unbound, exhibited similar distribution as evaluated by the Kolmorov-Smirnov test (*P*-value ≥ 0.05 in 96% of 200 resample rounds, with N_bound_=89 and N_unbound_=152 randomly-chosen values for each round)

None of these corrections affect the conclusion ‘the bending angle, before and after dsDNA encountered the vWF A1 domain, did not display statistically-significant differences’ or any other conclusion of the article.

