# Supplementary material for: DNA binds to a specific site of the adhesive blood-protein von Willebrand factor guided by electrostatic interactions
Source: Nucleic Acids Res. 2020 Jun 4;48(13):7333–44. doi: 10.1093/nar/gkaa466 (PMC7367192; doi:10.1093/nar/gkaa466)
Supplement: gkaa466_Supplemental_Files [file gkaa466_supplemental_files.zip › NAR-supp.pdf]

Supplementary Material of “DNA binds to a specific site of the adhesive blood-protein von Willebrand factor guided by electrostatic interactions”

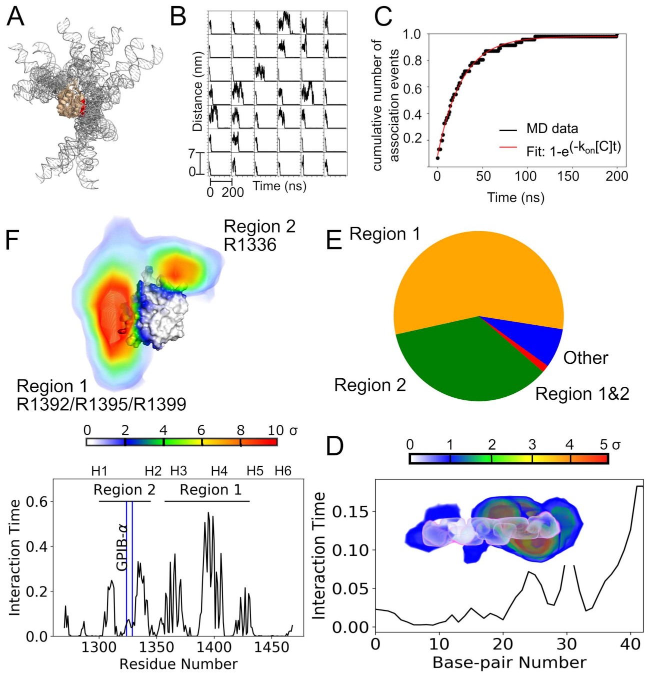

Figure S1. Spontaneous association of the vWF A1 domain to PolyAT ds DNA probed by equilibrium MD simulations. A. 15 representative conformations of the vWF A1-DNA encounter complex were superimposed, aligning the A1 domain to highlight the orientation variability of the bound ds DNA. Helix 4 (H4) of the A1 domain is highlighted in red. **B.** Minimum distance between the vWF A1 domain and the PolyAT as a function of time is presented. Each simulation corresponds to one of the stacked panels. **C.** Cumulative number of association events as a function of time at which the association took place (black) and a fit to the data (red) are displayed. Number of association events is normalized with respect to the total number of simulations $N = 42$. **D.** (Top) Time-average occupancy density of PolyAT ds DNA around the A1 domain is presented (color-coded according to color bar: sigma units with 0 corresponding to the background to the average density). (Bottom) The interaction time of each residue of A1 with the ds DNA is displayed. Location of the helices (H1 to H6) along the sequence is indicated. Reported binding site for the platelet receptor GPIB-α (1) is highlighted by the blue line. The main binding region (region 1) corresponds to the arginines R1392, R1395, and R1399 at helix H4 (compare higher interaction time and larger density for that region with that for the rest of the protein). A second binding region (region 2) consist of arginine R1336 (at the loop connecting the beta strand 3 and helix H2). **E.** Fraction of time the protein interacted with the ds DNA fragment is depicted: via R1392, 1395, and R1399 (Region 1: orange); R1336 (Region 2: green); both sets of arginines (regions 1 and 2: red), and other regions of the protein (blue). **F.** Interaction time of each base-pair of the ds DNA PolyAT fragment with the A1 domain. The inset shows the time-average occupancy protein density (blue to red) around the time-average occupancy DNA density (pink to white). The protein density is color-coded according to the shown color scale at different standard deviation (σ) units.

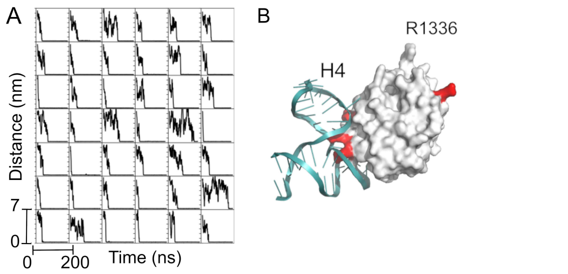

Figure S2. Spontaneous association of vWF A1 domain and ARC1172 ds DNA probed by equilibrium simulations. A. Minimum distance between the vWF A1 domain and the ARC1172 ds DNA as a function of time is presented. Each simulation corresponds to one of the stacked panels. **B**. The region predicted to bind ds DNA (red) agrees with the reported binding site for the single-stranded DNA aptamer ARC1172 (cartoon) (2).

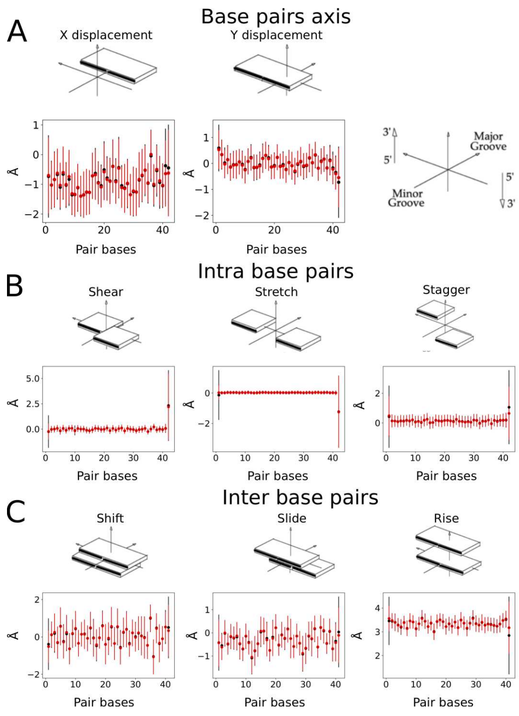

Figure S3. Internal translation dynamics of the ds DNA ARC1172 fragment. **A** Average displacements on X and Y axis, **B** intra and **C** inter base pairs spacing of the 42 base pairs before (red) and after (black) binding to vWF A1 (Average values are represented by points accompanied with the standard deviation bars).

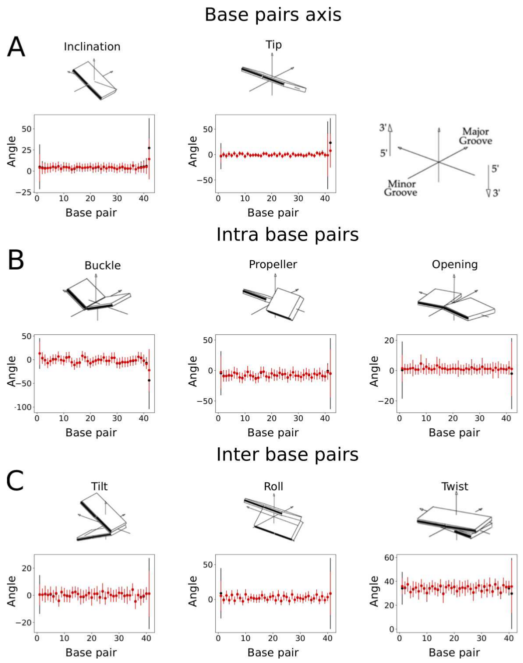

Figure S4. Internal rotation dynamics of the dsDNA ARC1172 fragment. **A** Average angle of inclination and Tip, **B** intra and **C** inter base pairs rotation angles for the 42 base pairs of the dsDNA ARC1172 fragment before (red) and after (black) binding to the protein vWF A1. (Average values are represented by points accompanied with the standard deviation bars).

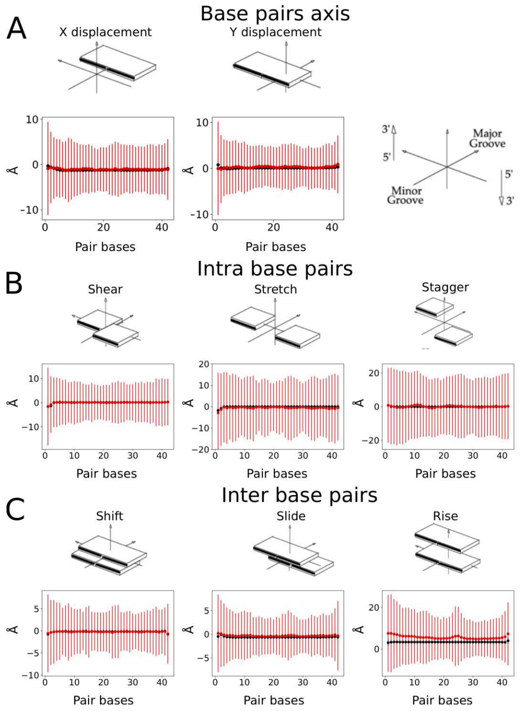

Figure S6. Internal translation dynamics of the ds DNA PolyAT fragment. **A** Average displacements on X and Y axis, **B** intra and **C** inter base pairs spacing of the 42 base pairs before (red) and after (black) binding to vWF A1. (Average values are represented by points accompanied with the standard deviation bars).

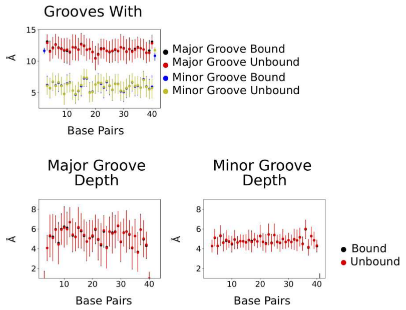

Figure S5. Average width and depth of Major and Minor Grooves of dsDNA ARC1772, previous and after binding to vWF A1.

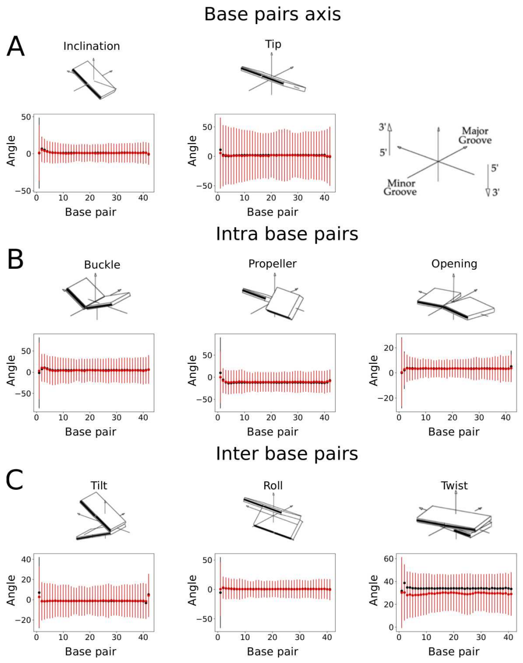

Figure S7. Internal rotation dynamics of the dsDNA PolyAT fragment. **A** Average angle of inclination and Tip, **B** intra and **C** inter base pairs rotation angles for the 42 base pairs of the dsDNA PolyAT fragment before (red) and after (black) binding to the protein vWF A1. (Average values are represented by points accompanied with the standard deviation bars).

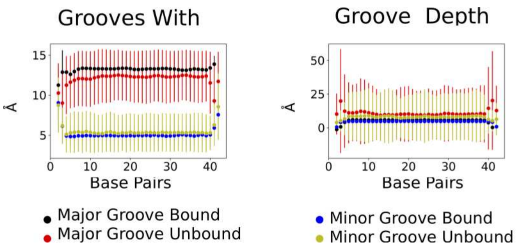

Figure S8. Average width and depth of Major and Minor Grooves of dsDNA PolyAT, previous and after binding to vWF A1.

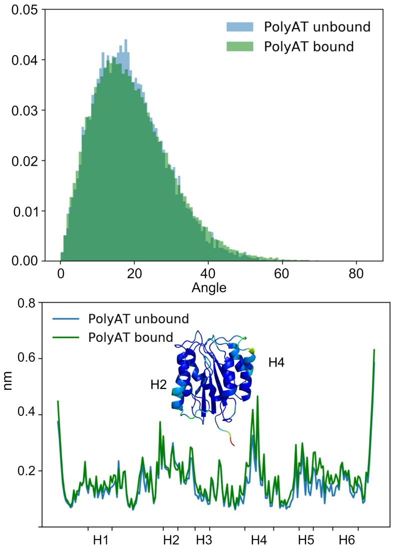

Figure S9. Internal dynamics of the PolyAT ds DNA fragment and vWF A1 domain, before and after association. *Top:* Bending angle distribution explored by ds DNA PolyAT before (blue) and after (green) binding to A1 is displayed. Both states, bound and unbound, exhibited similar distribution as evaluated by the Kolmogorov-Smirnov test (P-value>0.05 in 96% of 200 resample rounds, with $N_{\mathrm{bound}} = 89$ and $N_{\mathrm{unbound}} = 152$ randomly-chosen values for each round). *Down:* The root mean square fluctuation (RMSF) is presented for each amino-acid residue of A1 (location of the helices is indicated). Same color code as in the top panel. The inset depicts the location of helices H2 and H4 in cartoon representation.

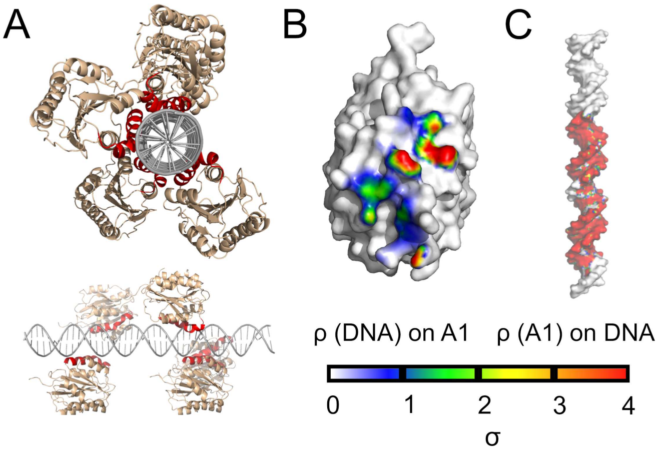

Figure S10. vWF A1–DNA interactions probed by rigid-body BD docking simulations. A Main docking conformations of the vWF A1 domain (wheat) around linear PolyAT ds DNA fragment recovered from rigid body BD docking simulations. Top and side views with respect to the ds DNA chain are shown. The helix H4 of vWF A1 is highlighted in red. Time-averaged occupancy density maps of ds DNA on A1, (**B.** ρ(DNA)), and of vWF A1 on ds DNA, (**C.** ρ(A1)), contoured at a surface 0.6 nm away from the respective molecule. The density is displayed according to the shown color-scale at standard deviation (σ) units, after normalizing the map. Accordingly, the average density here has a value of zero.

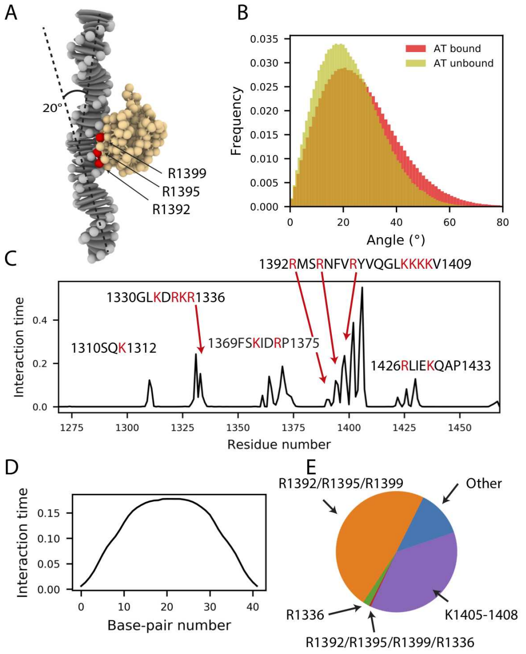

Figure S11. vWF A1–DNA polyAT interactions probed by CG simulations. A. Representative conformation of the vWF A1-DNA encounter, picturing the main bending angle that the dsDNA fragment adopted. **B.** The bending angle distribution in bound/unbound states. **C.** Fraction of interaction time in which the residues of the vWF A1 domain are in in contact with the polyAT ds DNA fragment. (0: if they were never in contact, and 1: if they were in contact all the time). **D.** Fraction of interaction time in which the base pairs are in contact with residues within the vWF A1 domain. **E.** Fractions of interaction time that ds DNA spent with the the arginines triad R1392/R1395/R1399, the arginine R1336, both sets or arginines simultaneously, the Lysine patch (residues 1405 to 1408) or other regions of the protein.

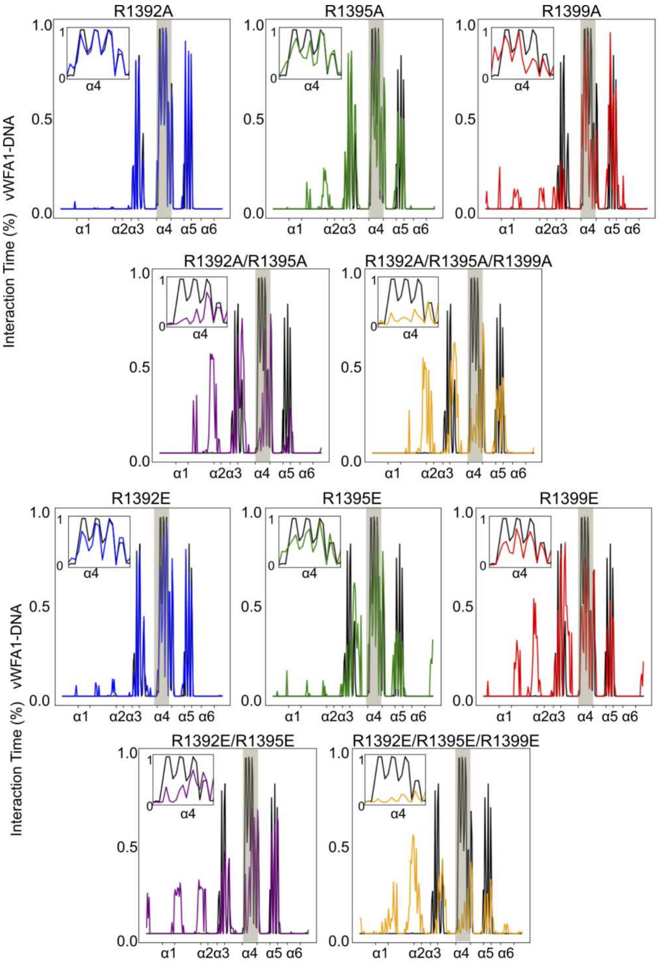

Figure S12. Total simulation time of A1 domain wt and mutations in close contact with the ds DNA fragment. Destabilization of the vWF A1-ds DNA interaction is spotted as reduced interaction time produced by the studied mutations (colored) in comparison with wt vWF A1 (black) bond to ds DNA.

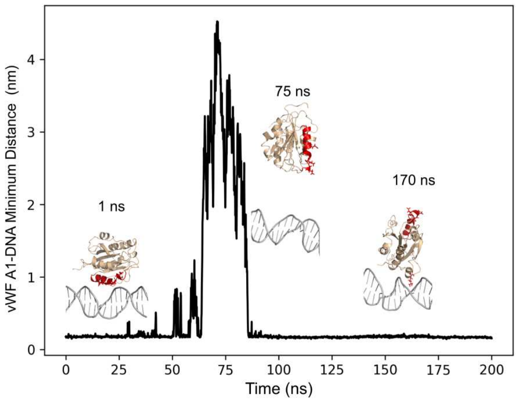

Figure S13. Dissociation of the vWF A1 domain from the ds DNA fragment was observed when the triple mutation R1392E/R1395E/R1399E was introduced. The distance between the mutated A1 domain and the ds DNA fragment is displayed as a function of time. Snapshots at the indicated times are displayed (A1: wheat; DNA: gray). The helix 4 (H4) containing the mutated arginines is highlighted in red. The dissociated vWF A1 domain binds later to ds DNA via the R1336 as shown in the snapshot at 170 ns.

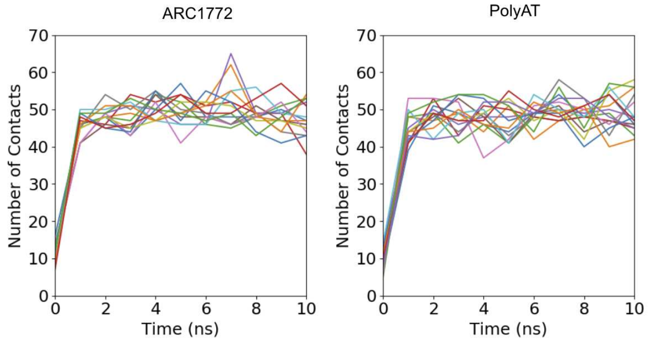

Figure S14. Number of contacts established between sodium ions and the ds DNA fragments ARC1772 and PolyAT, respectively, during 10 ns of equilibration previous to the MD production runs, is displayed. Each curve represents one simulation replica.

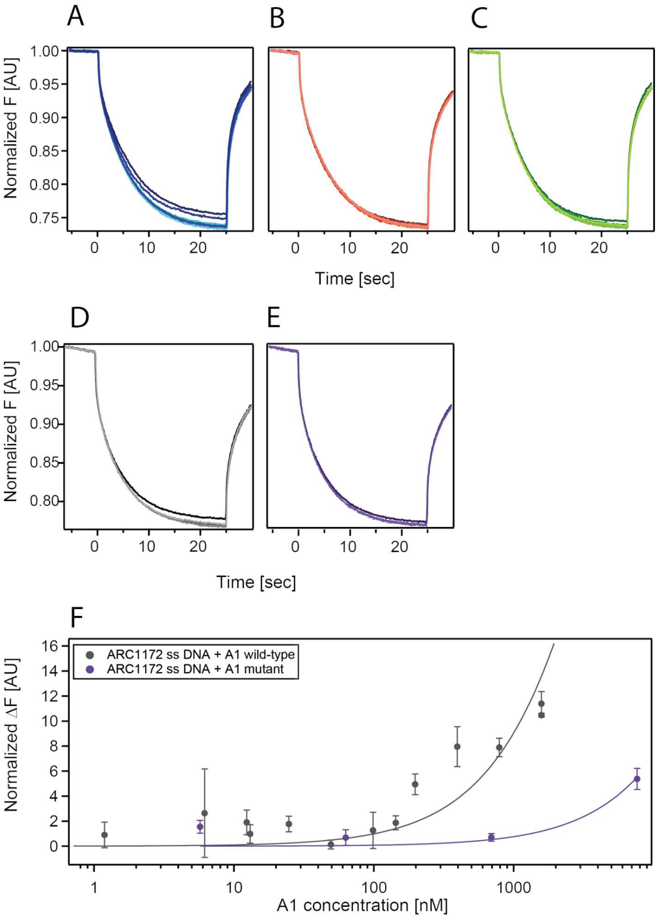

Figure S15. Change in normalized fluorescence (ΔF) recovered from thermophoresis experiments used to quantify binding of 23bp ds DNA fragments and ss DNA to single A1 domains. Titration curves correspond to ds DNA bound to **A.** the wt A1 domain in PBS buffer (blue), **B.** the A1 domain with mutations R1392A, R1395A and R1399A (green), and **C.** the A1 domain in PBS with additional 150 mM NaCl (grey). Additionally, titration curves for ss DNA bound to **D.** the A1 domain in PBS (grey), and **E.** A1 with mutations R1392A, R1395A and R1399A (purple). Titration curves were color-coded by A1 domain concentration from dark (high) to bright (low). **F.** Change in normalized ΔF for ARC1172 ss DNA and A1 domain (blue) with fit $Keq = 10 \pm 2\ \mu M$ and the A1 domain with mutations R1392A, R1395A and R1399A (green) with fit $Keq = 130 \pm 7\ \mu M$.

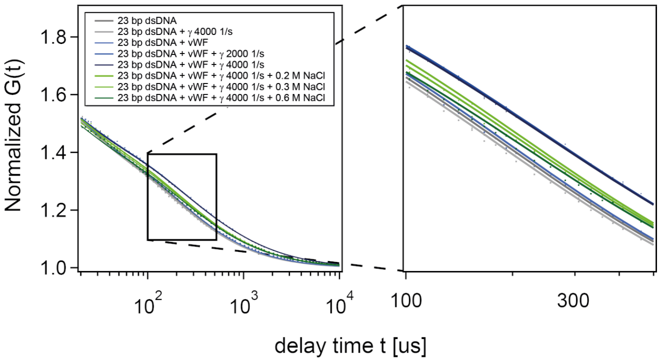

Figure S16. Binding of the wt vWF to random 23bp ds DNA under shear and different buffer conditions was measured by fluorescence correlation spectroscopy (FCS). Normalized autocorrelation functions for the 23bp ds DNA alone (grey) and after shearing (light grey). Blue curves correspond to samples with ds DNA and vWF: no shearing (light blue) and after different shearing rates (blue: 2000 1/s, dark blue 4000 1/s). ds DNA and vWF samples after addition of different salt concentrations (light green: 0.2 M NaCl, green: 0.3 M NaCl and dark green 0.6 M NaCl).

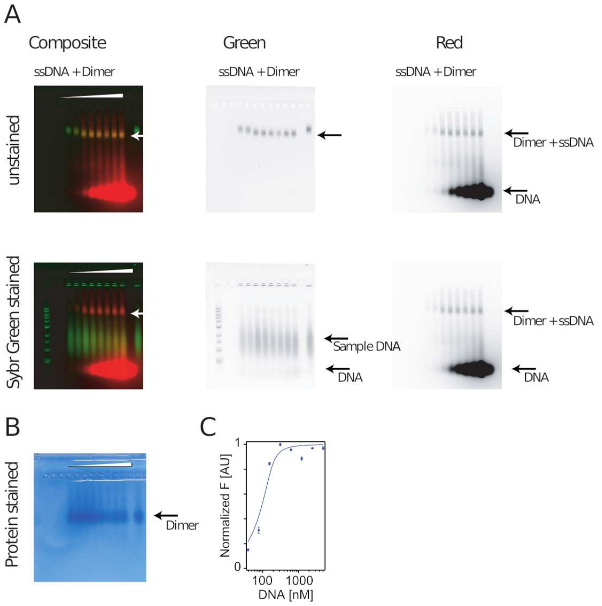

Figure S18. ss DNA binding to vWF dimers monitored by eletrophoretic mobility shift assays (EMSA). A. EMSA for different concentrations of ARC 1172 42 bp ss DNA (c(Dimer) = 0.47 mg/ml). Composite is an overlay of green (eGFP-Dimer or Sybr Green) and red (Cy5 DNA) channel. 1:1 titration of ss DNA from 5 μM to 20 nM. Ctrl DNA contains 500 nM Cy5 labelled DNA. **B.** Protein stained gel. **C.** Normalized Fluorescence for different concentrations of ss DNA at Dimer band with fit for $Keq = 1 \pm 6\, nM$.

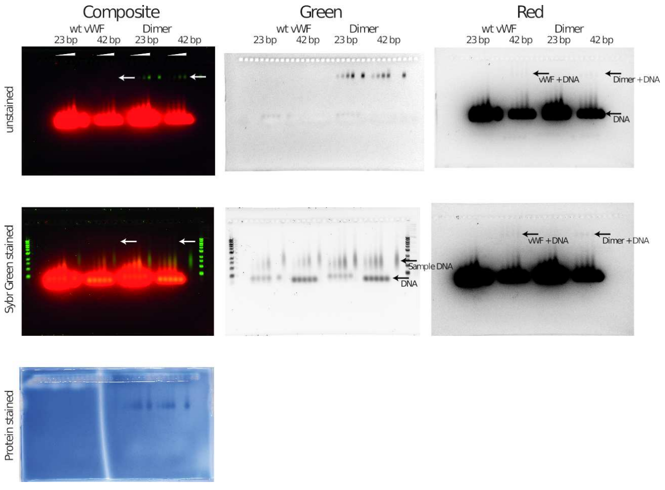

Figure S17. Comparison of DNA with different length binding to wt vWF or Dimer by EMSA. For the 23 bp DNA, no binding to vWF or dimer was observable, but for the 42 bp DNA for wt VWF as well as dimer a red signal was observable at the vWF/dimer band. EMSA for different concentrations of wt vWF or full length dimer binding to 42 bp DNA (c(DNA) = 300 nM). Composite is an overlay of green (eGFP-dimer or Sybr Green) and red (Cy5 DNA) channel. 1:1 titration of vWF from 130 μg/ml to 4 μg/ml. Ctrl DNA contains 300 nM Cy5 labelled DNA. 1:1 titration of Dimer from 313 μg/ml to 10 μg/ml. Blue image shows the protein stained gel.

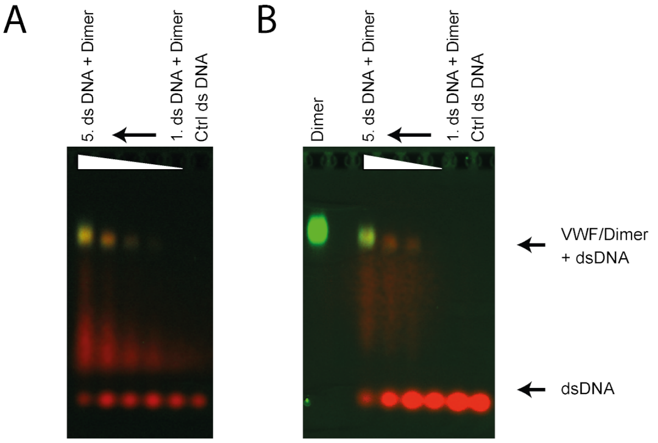

Figure S19. ds DNA binding to vWF dimers monitored by EMSA at different ionic strength. A. 30 mM and **B.** 60 mM.

SUPPLEMENTARY TEXT

S1. SUPPORTING MATERIALS AND METHODS

Starting Conformations

Starting conformations of the A1, A2, and A3 vWF domains were taken from their X-ray crystallographic structures (PDB ID codes: 3HXO (2), 3ZQK (3), and 1AO3 (4) for A1, A2, and A3, respectively). The ds DNA was assumed to have a linear starting conformation. Such conformation was generated using the make-na server (http://structure.usc.edu/make-na). Two 42-base-pair sequences were considered: poly adenine-thymine (PolyAT) and ARC1172, an aptamer experimentally optimized to bind the vWF A1 domain (GGC GTG CAG TGC CTT CGG CCG TGC GGT GCC TCC GTC ACG CCT) (2).

Atomistic Molecular Dynamics (MD) Simulations

Spontaneous Association of vWF A1 to ds DNA. Spontaneous association of vWF A1 with ds DNA, either to PolyAT or to ARC1172 was monitored using atomistic MD simulations. The ds DNA fragment was located at a distance at least 6.0 nm from the vWF A1 domain with 14 different relative orientations (Figure 1A of main text). All the simulations were performed using the package GROMACS, version 2016.3 (5). The following combination of force fields was chosen: Amber99sb-ILDN (6) was used for the protein, ParmBSC1 (7) for nucleic acids, Dang parameters (8) for the ions, and the TIP3P model (9) for the water molecules. First, the potential energy of the protein and the ds DNA were minimized during 50000 steps in vacuum using the steepest descent algorithm (10). Second, ≈142900 water molecules, ions at concentration 0.15 M, and counter-ions to neutralize the system were added, in a cubic box, of unit cell dimensions 15x15x20 nm^3, for a total of ≈436000 atoms. The solvated system was energy minimized for 50000 steps (also with the steepest descent algorithm). Three replicas were generated from this minimized system (for each of the 14 orientations). The solvent was equilibrated in each replica during 10 ns, by maintaining the protein and ds DNA positioned-restrained with harmonic springs on the heavy atoms (elastic harmonic constant of 1000 kJ/mol/nm^2). The position restraints were removed and production runs of 210 ns per replica were performed. Overall, the cumulative simulation time for both ds DNA fragments was 17.6 μs.

All the simulations were performed in the NPT ensemble, coupling the system to the Berendsen barostat (11) at a reference pressure of 1 bar and a coupling constant of 1 ps. The temperature was kept constant at 310 K, separately for the protein and ds DNA biomolecules and for the water and ions, using the Nose-Hoover thermostat (12, 13) (time constant of 0.5 ps). The center of mass of the system was removed every 100 steps. The long range electrostatic interactions were treated with the particle mesh Ewald technique (14, 15), in the real space for distances below 1.0 nm and in the reciprocal space beyond these distances. Short-range interactions were considered through a Lennard-Jones potential within a cut-off distance of 1 nm. Non-bonded neighbors were assigned through the Verlet buffer scheme (16). Bonds involving hydrogen atoms were constrained using the LINCS algorithm (17), and bonds and angles of water molecules were treated with SETTLE (18), hence allowing the integration of equations of motion at discrete time steps of 2 fs.

Equilibrium Simulations of the vWF A1–ds DNA Complex. MD simulations of the ARC1172 ds DNA fragment in complex with the vWF A1 domain were conducted, starting from a conformation predicted to have the highest interaction energy in Brownian dynamics (BD) simulations at a physiological salt concentration of 0.15 M. MD simulations were also carried out for ten vWF A1 mutants in complex with such a fragment. The arginines R1392, R1395, and R1399, located in the helix 4 of A1, were replaced by alanine or glutamic acid, resulting in single-, double-, or triple-amino acid mutations: R1392A(E), R1395A(E), R1399A(E), R1392A(E)/R1395A(E), and R1392A(E)/R1395A(E)/R1399A(E). Mutations were introduced in the protein with PyMol (19). Three simulation runs, of 200 ns each, were conducted for the wild-type (wt) complex and for each mutant, yielding an accumulated simulation time of 6.6 μs. The same simulation algorithms and parameters, as well as equilibration protocol, were used as in the MD simulations of spontaneous association (see above).

Analysis of atomistic MD simulations. The association process was monitored over time and an encounter complex was assumed to be established when the distance between vWF A1 and the ds DNA fragment was at most 0.6 nm. Cumulative histograms of the association times were computed by using the 42 MD replicas for each ds DNA fragment.

The time-average occupancy of ds DNA on vWF A1, and vice-versa, was calculated using the tool set Gromaρs (20). In brief, time-average 3-dimensional density maps were generated around each of these molecules considering conformations (from all 42 replicas) in which vWF A1 and the ds DNA fragments were in contact. Removal of the rigid-body translations and rotation preceded this calculation. For the case of the density of ds DNA around vWF A1, the conformations of the backbone atoms A1 were superposed by least square-fitting of their positions. For the case of the density of A1 around ds DNA, given the flexibility of the latter, the minVar package was used by applying the nearest neighbors method (min Var+NN), with ten iterations (21). The density was computed on a grid of 0.5 nm of resolution (along each coordinate), and the grid size was sufficiently large to convert the whole extension of the conformation ensemble of the two interacting biomolecules. Atomic densities were spread on the grid using a linear superposition of Gaussians, with their amplitudes and widths taken from (20).

The internal dynamics of vWF A1 and the ds DNA fragments PolyAT and ARC1172 was monitored throughout the simulation, distinguishing associated from dissociated conformations. On one hand, the flexibility of the vWF A1 protein was assessed by computing the root mean square fluctuation (RMSF) of each residue. On the other hand, the bending angle of the ds DNA fragments was computed. Subsequently, the normalized probability distribution of this angle was obtained. The distributions of the bending angle before and after association were compared using the two-sample Kolmogorov-Smirnov test (22). The test was carried out for a subset of N_{bound} and N_{unbound} uncorrelated angle values which were randomly-chosen from the angle time traces, in the bound and unbound states, respectively. To avoid correlation between chosen data points, the values were separated by a time window of at least 10 to 100 ns (time in which the autocorrelation function of the angle traces diminished from 1 to 0.1), thus yielding sample sizes between 23 and 152. The test was repeated 200 times considering different N_{bound} and N_{unbound} values and the number of cases the p-value was larger than 0.05 was quantified. In addition, local dynamic properties of the DNA were monitored using Curves+ (23). These properties include base-pair, inter base-pair, and intra base-pair translational and rotational motions, and width and depth of major and minor grooves. The time ds DNA spent interacting with each residue of A1 was analyzed (cutoff distance for 0.6 nm to assume an interaction). Both simulation sets, i.e.association simulations and those starting from the complex formed by these two molecules, were used for this purpose. In the association simulations, the interaction time was only monitored when DNA was bound to A1. The interaction time was normalized according to the total number of snapshots in which A1 and the ds DNA fragment were in contact. In a similar fashion, the time A1 spent interacting with each base pair on the DNA was also monitored.

Atomistic rigid-body Brownian Dynamics (BD) simulations

Docking of vWF A domains to ds DNA. The vWF A1, A2, and A3 domains were docked to both the ds DNA ARC1172 and the PolyAT fragments, using rigid-body BD simulations. Polar hydrogen atoms were added using PDB2PQR tool (24). Partial atomic charges and van der Waals parameters were taken from the AMBER99 force field (25). The electrostatic potential was calculated for all the structures using non-linear Poisson-Boltzmann equation with a grid spacing of 0.1 nm and dimensions of 0.129^3 nm^3 at a temperature of 300 K. The solvent and solute dielectric constants were set to 78 and 1, respectively. Note that previous BD simulations of histone–DNA interactions remained insensitive to changes in the solute dielectric constant from 2 to 8 (26), closer to our chosen value. An ionic strength corresponding to a salt concentration of 0.15 M was considered. In addition, a salt concentration of 1 M was also tested for the system consisting of the vWF A1 domain and the ds DNA ARC1172 fragment.

BD docking simulations were performed with the package Simulation of Diffusion and Association (SDA, version 7) (27). Electrostatic interactions were considered by representing each molecule with a small number of effective charges in a uniform dielectric medium (28). Soft-core repulsion was neglected; instead, an exclusion grid of 0.05 nm was used to avoid overlapping.

The structures were located randomly, in the three-dimensional space, at a starting center of mass distance between the ds DNA fragment and the vWF A1 domain, d_{start}, listed in Table S1. A scheme involving multiple time-integration steps was considered, in the same fashion as described by Öztürk *et al.* (26). For distances larger than the value $d_{\Delta t=20ps}$, the time step was set to 20 ps. For distances between $d_{\Delta t=20ps}$ and $d_{\Delta t=1ps}$, the time step decreased linearly from 20 ps down to 1 ps. For distances smaller than $d_{\Delta t=1ps}$, the time step was maintained constant at 1 ps (all threshold distances used are summarized in Table S1). A total of 20000 docking runs were generated for each case. Complex formation was assumed to occur, when the center-of-mass distance between both molecules was lower than d_{end} and the surface to surface distance was zero. The structure of the complex was recorded if, compared to the previously recorded complex, the RMSD was lower than 0.1 nm and the potential energy was lower. In total, 500 poses of the complex were collected. The predicted poses were clustered in five groups based on their RMSD similarity (clustering cutoff of 0.3 nm). For each cluster, one representative pose was selected, and the following quantities were extracted: size in number of poses and average interaction energy (see Table S2).

Association of vWF A1 to ds DNA ARC1172. To estimate the association kinetic rate, k_{on}, a set of BD association simulations were performed. Partial charges, van der Waals parameters, threshold distances, and other simulation parameters were kept equal as in the docking procedure. 20000 association trajectories were produced. On one hand, the simulations were terminated when the center-of-mass to center-of-mass distance exceeded a value of 40 nm, without association events. On the other hand, the complex formation was defined when four independent contacts were sampled at a distance of 0.6 nm. If more than one residue established an interaction with ds DNA, and these residues were within a radius of 0.6 nm, the contact was counted as one. For distances longer than 0.6 nm between residues, the interactions were counted as independent. The Northrup-Allison-McCammon method to calculate the association rate constant k_{on} was applied (29).

Coarse-Grained (CG) MD Simulations.

Spontaneous association of vWF A1 to ds DNA (PolyAT or ARC1172) was further explored using CG MD simulations. We performed 64 simulations (32 repeats with different random number generator seeds for each ds DNA sequence).

Table S1. Summary of the threshold distances, considered during the BD docking simulations for the indicated vWF A domains (all distances are in nm).

Protein Domain	d_{start}	$d_{\Delta t=1ps}$	$d_{\Delta t=20ps}$	d_{end}
A1	30.14	20.85	22.75	9.36
A2	29.85	20.23	22.13	7.63
A3	29.85	20.35	22.55	4.03

For each simulation trajectory, the DNA was assigned a random initial position, configuration, and orientation that kept it a minimum distance of 2 nm from the vWF A1. Each trajectory of 3.5 μs was run in the NVT ensemble using a Langevin thermostat set at 300 K with a relaxation time of 100 ps and a time step of 10 fs. All simulations were performed at monovalent salt concentration of 0.15 M in a cubic box of $40^3 nm^3$ using the LAMMPS MD package (30) .

The A1 domain CG model was designed to preserve the shape, secondary structure, and amino acid charge distribution of the protein. The experimentally determined tertiary structure (2) worked as a reference to map each amino acid into a spherical bead. The center of each bead was placed at the geometric center of each C_α in the vWF A1. Additionally, the protein structure was preserved by imposing an elastic network, which joined the beads within a cut-off of 0.7 nm with a semi-rigid harmonic potential of 10 kcalÅ^{-2}mol^{-1} and equilibrium distances, taken from the reference structure. The total charge of the amino acids was assigned to the corresponding beads; i.e., both Arginine and Lysine have a total charge of $+1e$.

Both ds DNA fragments were coarse-grained using the rigid base-pair model (31, 32, 33), which computes the DNA mechanical potential energy (V_M^{DNA}) from the sum of harmonic distortions of equilibrium base-pair step geometries in terms of the helical parameters:

$$V_M^{\mathrm{DNA}} = \frac{1}{2}(\psi - \psi_0)^{\mathrm{T}} \cdot \mathbf{K} \cdot (\psi - \psi_0) \quad (1)$$

where ψ is the 6-dimensional vector of the helical parameters (twist, roll, tilt, slide, shift, and rise) that accounts for the relative positions and orientations between neighboring basepair planes. For the vector of equilibrium basepair configurations, ψ_0, and the 6 x 6 stiffness matrix, $\mathbf{K}$, we use the sequence-dependent parameters at the NAFlex webserver (34). We explicitly include salt-dependent electrostatic interactions and excluded volume to describe the DNA-protein interactions, by placing a bead at the center-of-mass position of each phosphate. This additional bead carries a charge of -1e and an excluded volume term to avoid overlap with the protein beads.

Protein-charged-beads interact via Debye Hückel potential with the DNA beads, which allows us to approximate screening by counterions in solution:

$$V_{ec}^{prot-DNA} = \frac{q_i q_j}{4\pi\epsilon_0\epsilon_r r} e^{-r/\lambda_d} \quad (2)$$

where q_i and q_j are the charges of the beads and DNA phosphates. ϵ_0 is the vacuum permittivity and ϵ_r is the relative permittivity of the medium (80 for water). λ_d is the screening length which is 0.8 nm for 0.15 nM. Besides, we use the following truncated and shifted Lennard-Jones potential to account for the excluded volume of each residue and avoid overlap between protein and DNA beads.

$$V_{ev}^{prot-DNA} = \begin{cases} 4\epsilon\left[\left(\frac{\sigma}{r}\right)^{12} - \left(\frac{\sigma}{r}\right)^{6}\right] + \epsilon, & \text{for } r \le 2^{1/6}\sigma \\ 0, & \text{for } r > 2^{1/6}\sigma \end{cases} \quad (3)$$

where σ, ϵ are 0.4 nm, 0.1 kcal/mol for protein-phosphate and 0.8 nm, 0.01 kcal/mol for protein-DNA basepair.

Samples

Full-length vWF and domain constructs. The mammalian pIRESneo2 expression vector containing the cDNA of full-length wt vWF was generated as previously described (35). To express and purify the single A1 domain, the coding sequences of domains A1-A2 (aa 1230–1672) carrying the N-terminal signal peptide of vWF was cloned into pIRESneo2 (NheI + NotI). Employing the QuikChange Multi Site-Directed Mutagenesis Kit (Agilent), a His6-tag and a furin cleavage site (ARG-Ser-Lys-Arg) were inserted between A1 and A2. Plasmids pIRESneo2-vWF and pIRESneo2-vWF-A1-His-furinsite-A2 were further used for mutagenesis using the QuikChange Multi Site-Directed Mutagenesis Kit (Agilent) to insert indicated single mutations Arg1392Ala, Arg1395Ala and Arg1399Ala or combination of these mutations. All primers are available upon request.

Cleavage by endogenous furin within the expressing HEK293 cells does not yield 100% cleaved A1. Thus all his-tagged A1 and A1-A2 proteins were purified employing the His-Pur Ni-NTA Resin (Thermo Fisher Scientific) according to the manufacturer's instructions using a gravity-flow column. Eluates were pooled and dialysis was performed in 100 mM HEPES, 1 mM CaCl2, pH 7.4. Afterwards, 0.5% triton X-100 (Sigma-Aldrich), 1 mM 2-mercaptoethanol (Sigma-Aldrich) and 2 U/ml Furin (Biolabs) were added to the dialyzed samples and incubated shaking at RT for 16 h. The completely cleaved samples were then purified again via the His-tag as described above.

DNA. In the thermophoresis and FCS experiments two different ds DNA duplexes were tested, one of 23bp and other of 42bp in length, respectively. The 23bp ds DNA was purchased by Eurofins Genomics Germany GmbH consisting of the sequences:

1. CAT AAA TCT TTG AAT ACC AAG TGT TAG AAC CAT CAA CCA TAT CAA CTT CCT TTA TAC ATC TA
2. CTA CAT CAC TTT CTT CAT TAT AAA [CY5]AT AAA AGG AAG TTG ATA TGG TTG ATG

The 42bp ARC1172 DNA was purchased by Integrated DNA Technologies, Inc.:

3. AGG CGT GAC GGA GGC ACC GCA CGG CCG AAG GCA CTG CAC GCC
4. GGC GTG CAG TGC CTT CGG CCG TGC GGT GCC TCC GTC ACG CCT AAA/iCy5/ATA AAA

For the ss DNA only the labeled strand was used. Concerning the ds DNA samples, both ds DNA samples were annealed in 12 mM MgCl2 1x TAE buffer for 2 h with a temperature ramp from 90 °C to 4 °C at a concentration of 10 μM and kept frozen until used.

Thermophoresis Experiments

The vWF A1 domains were purified by applying four wash steps with a 10 K Amicon Ultra Centrifugal Filter to remove Imidazole from the sample. Subsequently, the vWF A1 domain concentration was determined with a NanoDrop TM 1000 Spectrophotometer and titrated with 1:1 or 3:1 dilution in phosphate buffered saline (PBS) and then mixed 1:1 with Cy5-DNA premix of 20 nM yielding 10 nM ds DNA concentration. After 10 min of incubation, samples were loaded to NT.115 MST standard capillaries and measured with a Monolith ® NT.115 Pico instrument (NanoTemper Technologies) at 22°C with 2% light-emitting diode (LED) and 40% infrared (IR) powers with IR laser on/off times of 25 and 5 s, respectively. Each capillary was measured four times and normalized. The change in normalized fluorescence ΔF was background subtracted to the lowest value in the unbound state and all data was batch fitted in Igor Pro 7 with the general binding isotherm:

$$\Delta F(C_{A1}) = 100*0.5(K_{eq}+C_{A1}+C_{DNA}- \frac{\sqrt{(K_{eq}+C_{A1}+C_{DNA})^2-4C_{A1}C_{DNA})}}{C_{DNA}},$$

with K_{eq} being the dissociation constant, C_{A1} and C_{DNA} the concentration of the A1 domain and ds DNA, respectively.

Fluorescence Correlation Spectroscopy (FCS)

DNA binding to vWF was studied with FCS on an Axiovert 200 microscope with a ConfoCor 2 unit, equipped with a water-immersion 40x (NA=1.2) Apochromat objective (Carl Zeiss, jena, Germany). For illumination a 633 HeNe Laser was operated. The system was calibrated with a Cy5 or Alexa 633 dye in the corresponding buffer for 10×30 s in the shear cell chamber (36, 37), which was previously passivated with ultra-heat treated milk for 1 h and washed. Subsequently, 1 nM ds DNA (23 bp or 42bp) sample alone was characterized before and after shearing with 2000 s^{-1} or 4000 s^{-1}, respectively. Afterwards, a sample with 300 nM vWF and 1 nM ds DNA was investigated, first without shearing and then after shearing for 5 min with 2000 s^{-1} and 4000 s^{-1}. To investigate the effect of salt, 5 M NaCl was added to achieve final concentrations of 150 mM - 600 mM.

Electrophoretic Mobility Shift Assays (EMSA)

Samples were prepared in TA buffer and incubated on a shaker for 1 h. Afterwards, the samples were loaded on a 0.75 percentage agarose gel and run cooled and shielded from light in TA buffer at 70 V for 2 h. Subsequently, the gel was imaged on a Biorad Chemidoc MP equipped with 530/28 and 695/55 filter sets to evaluate the blue or red Epi illumination of the gels. Afterwards, the gel was stained with SybrGreen dye purchased from ThermoFisher and imaged again with the same settings. Then the gel was stained with the PageBlue Protein Staining Solution and imaged again. The gels were run in a buffer with 40 mM ionic strength for the 43 bp long ss DNA. To test the effect of the ionic strength, for the 43 bp ds DNA, binding 0.5 TA (20 mM) buffer was supplemented with 10 mM and 40 mM NaCl to obtain overall ionic strengths of 20, 30, and 60 mM.

Microfluidic Experiments

For microfluidic experiments, we used a pneumatically driven channel system (BioFlux, San Francisco, CA, USA). Channels were coated with full-length wt vWF or full-length vWF with the mutation R1399A in the A1 domain (R1399A vWF). For the functional characterization of vWF-ds DNA interactions under flow conditions, channels were mounted onto an inverted fluorescence microscope (Zeiss Axio Observer Z.1, Carl Zeiss, Jena, Germany) and perfused with a solution containing 15 μg/ml ds DNA (lambda DNA cI857Sam7, ThermoFisher Scientific, Passau, Germany), stained with 4',6-diamidino-2-phenylindole (DAPI), and 20 μg/ml wt vWF or R1399A vWF. Here, we chose a shear rate of 4000 s^{-1} to ensure a fully-stretched active conformation of free-floating vWF (38). By considering the viscosity of the perfusion medium, this shear rate corresponds to a shear stress of 40 dyn/cm^2, a typical value found in the arterial flow path (39). After lowering the shear rate to 100 $^{-1}$, time-lapse fluorescence images were taken with 1.25 frames/s and analyzed by 'aggregate motion tracking' using ZEN software (Carl Zeiss, Jena, Germany) and the open-source software ImageJ (40) as previously published (41).

S2. BROWNIAN DYNAMICS SIMULATIONS OF THE VWF A2 AND A3 DOMAINS

BD simulations also allowed comparison of the binding of ds DNA to other vWF A domains, namely A2 and A3. In the BD docking simulations, the interaction energy of the best-ranked clusters was considered indicative of the strength of the interaction. The docking of A1 to ARC1172 and PolyAT sequences resulted in interaction energies of $-48.4\pm0.4\ k_BT$ and $-46.6\pm0.2\ k_BT$, respectively (with k_B the Boltzmann constant and T the temperature). If instead of A1, both A2 and A3 domains were docked, the strength of interaction was substantially weakened to values of $-3.4\pm0.2\ k_BT$ and $-20.9\pm0.2\ k_BT$, respectively (Table S2). In addition, the size of the five most representative clusters was drastically reduced for the A2 and A3 domains compared to the A1 domain (Table S2). Moreover, it was found that ds DNA bound to different regions on the A2 and A3 domains, contrary to the specific helix targeted in the case of A1 (Figure S20). This comparison highlights A1, among the three vWF A domains, as the main site responsible for the interaction with ds DNA, as previously suggested experimentally (38).

A1–ARC1172

Cl#	ClSize	ClFSize	ClAE	CLAED
1	297	491963	-48.4	0.4
2	104	68647	-48.1	0.2
3	41	39314	-47.9	0.2
4	40	162603	-47.8	0.2
5	18	15796	-47.8	0.1

A1–PolyAT

Cl#	ClSize	ClFSize	ClAE	CLAED
1	195	169545	-46.6	0.2
2	148	277851	-46.6	0.2
3	89	98257	-46.6	0.2
4	43	16048	-46.5	0.1
5	25	25283	-46.6	0.1

A2–ARC1172

Cl#	ClSize	ClFSize	ClAE	CLAED
1	160	915	-3.4	0.2
2	113	564	-3.3	0.2
3	102	543	-3.3	0.2
4	79	414	-3.4	0.3
5	45	353	-3.4	0.2

A3–ARC1172

Cl#	ClSize	ClFSize	ClAE	CLAED
1	277	7572	-20.9	0.2
2	116	2443	-20.8	0.2
3	78	1881	-20.8	0.1
4	24	423	-20.9	0.2
5	4	140	-20.690	0.032

Table S2. Population statistics of clusters from Brownian dynamics rigid-body docking simulations for the indicated vWF A–ds DNA binding is presented. ClSize: Number of entries in the cluster. **ClFSize:** Number of representative entries for the cluster. **ClAE:** Average total energy of all cluster members weighted by the number of representatives. **CLAED:** Weighted standard deviation of the total energy of the cluster. Energies are given in K_BT units, with K_B the Boltzmann constant and T the temperature.

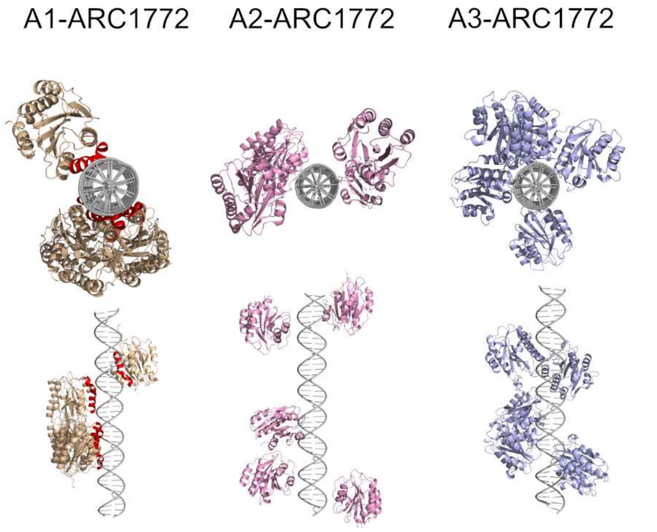

Figure S20. Binding of vWF A1, A2, and A3 domains to the ds DNA ARC1772 fragment probed by Brownian dynamics simulations. Representative conformations of the encounter complex established by ds DNA (gray) and the vWF A1 (wheat), A2 (pink), and A3 (light-blue) domains are depicted. For A1, the helix 4 (H4) is highlighted in red.

S3. DEBYE-HÜCKEL APPROXIMATION

To elucidate the origin of the fast time-scales involved, as a first approximation, the Debye-Hückel theory was employed. The magnitudes of the electrostatic interaction energy, $\psi(r)$, and the force, F_{el}, between these two charges at a distance r read:

$$\psi(r) = \frac{q_{A1} q_{ARC1172}}{4\pi\epsilon_0\epsilon_r} \frac{e^{\kappa a_0}}{1+\kappa a_0} \frac{e^{-\kappa r}}{r}, \qquad (4)$$

$$F_{el} = \frac{q_{A1} q_{ARC1172}}{4\pi\epsilon_0\epsilon_r} \frac{e^{\kappa a_0}}{1+\kappa a_0} \frac{e^{-\kappa r}}{r^2} \left(1+\kappa r\right) \qquad (5)$$

Here, ϵ_0 is the vacuum permitiivity, ϵ_r is the relative permittivity (assumed here to be 80), a_0 is the closest distance that ions can approach the two charges (here about the size of A1 and the ds DNA fragment) and $1/\kappa$ the Debye screening length. The latter term takes into account the ionic concentration of the solution, C, as follows: $\kappa^2 = (2N_A e^2 C)/(\epsilon_0\epsilon_r k_B T)$, with N_A the Avogadro number and e the elementary charge. Let us focus on A1. This domain experiences an average viscous force due to the surrounding aqueous medium of magnitude $F_{vis} = (k_B T/D)(dr/dt)$, where dr/dt is the speed at which this domain approaches the ds DNA fragment and D its diffusion coefficient. In the diffusive regime, when the friction is so strong that velocities relax almost instantaneously, the electrostatic and the viscous forces cancel each other out: $F_{el} = F_{vis}$. Thus, A1 moves at a speed $dr/dt = (D/k_B T)F_{el}$. From this equation, a characteristic time τ to move from an initial distance d_0 to a contact distance d_c can be estimated as:

$$\tau = \frac{k_B T}{D} \int_{d_0}^{d_c} \frac{1}{F_{el}(r)} dr \qquad (6)$$

By using $\epsilon_r = 80$, $a_0 = 3.61$ nm, $C = 0.15$ M, $d_0 = 7$ nm, $d_c = 3.61$ nm and $D = 1.9\ nm^2/ps$ (the latter estimated from the diffusional motion of A1 before entering into contact with DNA), a numerical solution of equation **6** assuming increasing force while the particles approach (Figure S21), yields an encounter time of $\tau = 34$ ns, which agree with the fast encounter vWF A1-DNA by MD simulations.

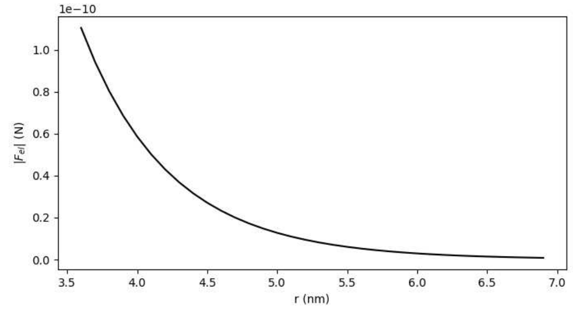

Figure S21. The magnitude of the interaction force F_{el} between two point charges $q_{A1} = +6e$ and $q_{DNA} = -82e$, predicted by Debye-Hückel theory (see equation 5), is depicted as a function of the separation r between the charges.

MOVIE S1 CAPTION

Binding of full-length vWF to ds DNA under shear flow conditions was measured in microfludic experiments. To characterize shear dependent interactions of ds DNA, stained with DAPI, to wild-type vWF (upper channel) in comparison to vWF with the mutation R1399A in the A1 domain (lower channel), a 20 s real time sequence (taken at a frequency of 1.25 images per s) of a representative microfluidic experiment is displayed. The movie has been started after a high shear application pretreatment with a shear rate of 4000 1/s for 60 s. The white bar corresponds to 100 μm.

REFERENCES

1. Dumas, J., Kumar, R., McDonagh, T., Sullivan, F., Stahl, M. L., Somers, W., and Mosyak, L. (2004) Crystal structure of the wild-type von Willebrand factor A1-Glycoprotein Ib-α complex reveals conformation differences with a complex bearing von Willebrand disease mutations. *J. Biol. Chem.,* **279**(22), 23327–23334.
2. Huang, R. H., Fremont, D. H., Diener, J. L., Schaub, R. G., and Sadler, J. E. (2009) A structural explanation for the antithrombotic activity of ARC1172, a DNA aptamer that binds von Willebrand factor domain A1. *Structure,* **17**(11), 1476–1484.
3. Jakobi, A. J., Mashaghi, A., Tans, S. J., and Huizinga, E. G. (2011) Calcium modulates force sensing by the von Willebrand factor A2 domain. *Nat. Commun.,* **2**, 385.
4. Bienkowska, J., Cruz, M., Atiemo, A., Handin, R., and Liddington, R. (1997) The von Willebrand factor A3 domain does not contain a metal ion-dependent adhesion site motif. *J. Biol. Chem.,* **272**(40), 25162–25167.
5. Abraham, M. J., Murtola, T., Schulz, R., Páll, S., Smith, J. C., Hess, B., and Lindahl, E. GROMACS: High performance molecular simulations through multi-level parallelism from laptops to supercomputers.. *SoftwareX,* **1-2**, 19 – 25.
6. Lindorff-Larsen, K., Piana, S., Palmo, K., Maragakis, P., Klepeis, J. L., Dror, R. O., and Shaw, D. E. (2010) Improved side-chain torsion potentials for the Amber ff99SB protein force field. *Proteins,* **78**(8), 1950–1958.
7. Ivani, I., Dans, P. D., Noy, A., Pérez, A., Faustino, I., Hospital, A., Walther, J., Andrio, P., Goñi, R., Balaceanu, A., Portella, G., Battistini, F., Gelpí, J. L., González, C., Vendruscolo, M., Laughton, C. A., Harris, S. A., Case, D. A., and Orozco, M. (2015) Parmbsc1: a refined force field for DNA simulations. *Nat. Methods,* **13**, 55–58.
8. Dang, L. X. (1995) Mechanism and thermodynamics of ion selectivity in aqueous solutions of 18-crown-6 ether: a molecular dynamics study. *J. Am. Chem. Soc.,* **117**(26), 6954–6960.
9. Jorgensen, W. L., Chandrasekhar, J., and Madura, J. D. (1983) Comparison of simple potential functions for simulating liquid water. *J. Chem. Phys.,* **79**(2), 926–935.
10. Berendsen, H. J. C. and Van Gunsteren, W. F. (1986) Practical algorithms for dynamic simulations, North-Holland, Amsterdam, .
11. Berendsen, H. J. C., Postma, J. P. M., van Gunsteren, W. F., DiNola, A., and Haak, J. R. (1984) Molecular dynamics with coupling to an external bath. *J. Chem. Phys.,* **81**(8), 3684–3690.
12. Nosé, S. (1984) A unified formulation of the constant temperature molecular dynamics methods. *J. Chem. Phys.,* **81**, 511–519.
13. Hoover, W. G. (1985) Canonical dynamics: Equilibrium phase-space distributions. *Phys. Rev. A,* **31**, 1695–1697.
14. Darden, T., York, D., and Pedersen, L. (1993) Particle mesh Ewald: An N log (N) method for Ewald sums in large systems. *J. Chem. Phys.,* **98**(12), 10089–10092.
15. Essmann, U., Perera, L., Berkowitz, M. L., Darden, T., Lee, H., and Pedersen, L. G. (1995) A smooth particle mesh Ewald method. *J. Chem. Phys.,* **103**(19), 8577–8593.
16. Páll, S. and Hess, B. (2013) A flexible algorithm for calculating pair interactions on SIMD architectures. *Comput. Phys. Commun.,* **184**(12), 2641–2650.
17. Hess, B., Bekker, H., Berendsen, H. J. C., and Fraaije, J. G. E. M. (1997) LINCS: a linear constraint solver for molecular simulations. *J. Comp. Chem.,* **18**(12), 1463–1472.
18. Miyamoto, S. and Kollman, P. A. (1992) Settle: An analytical version of the SHAKE and RATTLE algorithm for rigid water models. *J. Comp. Chem.,* **13**(8), 952–962.
19. Delano, W. L. (2002) The PyMOL Molecular Graphics System, available: http://www.pymol.org.
20. Briones, R., Blau, C., Kutzner, C., de Groot, B. L., and Aponte-Santamaría, C. (2019) Gromaps: a GROMACS-based toolset to analyse density maps derived from molecular dynamics simulations. *Biophys J.,* (1), 4–11.
21. Gapsys, V. and de Groot, B. L. (2013) Optimal superpositioning of flexible molecule ensembles. *Biophys. J.,* **104**(1), 196–207.
22. Lehmann, E. L. and D'Abrera, H. (1975) Nonparametrics: statistical methods based on ranks, Holden-Day, .
23. Lavery, R., Moakher, M., Maddocks, J. H., Petkeviciute, D., and Zakrzewska, K. (2009) Conformational analysis of nucleic acids revisited: Curves+. *Nucleic Acids Res.,* **37**(17), 5917–5929.
24. Dolinsky, T. J., Nielsen, J. E., McCammon, J. A., and Baker, N. A. (2004) PDB2PQR: an automated pipeline for the setup of Poisson–Boltzmann electrostatics calculations. *Nucleic Acids Res.,* **32**(suppl_2), W665–W667.
25. Wang, J., Cieplak, P., and Kollman, P. A. (2000) How well does a restrained electrostatic potential (RESP) model perform in calculating conformational energies of organic and biological molecules?. *J. Comp. Chem.,* **21**(12), 1049–1074.
26. Öztürk, M. A., Pachov, G. V., Wade, R. C., and Cojocaru, V. (2016) Conformational selection and dynamic adaptation upon linker histone binding to the nucleosome. *Nucleic Acids Res.,* **44**(14), 6599–6613.
27. Martinez, M., Bruce, N. J., Romanowska, J., Kokh, D. B., Ozboyaci, M., Yu, X., Öztürk, M. A., Richter, S., and Wade, R. C. (2015) SDA 7: A modular and parallel implementation of the simulation of diffusional association software. *J. Comp. Chem.,* **36**(21), 1631–1645.
28. Gabdoulline, R. R. and Wade, R. C. (1996) Effective charges for macromolecules in solvent. *J. Phys. Chem.,* **100**(9), 3868–3878.
29. Northrup, S. H., Allison, S. A., and McCammon, J. A. (1984) Brownian dynamics simulation of diffusion-influenced bimolecular reactions. *J. Chem. Phys.,* **80**(4), 1517–1524.
30. Plimpton, S. (1995) Fast Parallel Algorithms for Short-Range Molecular Dynamics. *J. Comput. Phys.,* **117**(1), 1 – 19.
31. Olson, W. K., Gorin, A. A., Lu, X., Hock, L. M., and Zhurkin, V. B. (1998) DNA sequence-dependent deformability deduced from protein–DNA crystal complexes. *Proc. Nation. Acad. Sci.,* **95**(19), 11163–11168.
32. Lankaš, F., Šponer, J., Langowski, J., and Cheatham, T. E. (2003) DNA Basepair Step Deformability Inferred from Molecular Dynamics Simulations. *Biophys. J.,* **85**(5), 2872 – 2883.
33. Pérez, A., Noy, A., Lankas, F., Luque, F. J., and Orozco, M. (01, 2004) The relative flexibility of B-DNA and A-RNA duplexes: database analysis. *Nucleic Acids Res.,* **32**(20), 6144–6151.
34. Hospital, A., Faustino, I., Collepardo-Guevara, R., González, C., Gelpí, J. L., and Orozco, M. (05, 2013) NAFlex: a web server for the study of nucleic acid flexibility. *Nucleic Acids Res.,* **41**(W1), W47–W55.
35. Müller, J. P., Mielke, S., Löf, A., Obser, T., Beer, C., Bruetzel, L. K., Pippig, D. A., Vanderlinden, W., Lipfert, J., Schneppenheim, R., and Benoit, M. (2016) Force sensing by the vascular protein von Willebrand factor is tuned by a strong intermonomer interaction. *Proceedings of the National Academy of Sciences,* **113**(5), 1208–1213.
36. Lippok, S., Obser, T., Müller, J. P., Stierle, V. K., Benoit, M., Budde, U., Schneppenheim, R., and Rädler, J. O. (2013) Exponential size distribution of von Willebrand factor. *Biophys. J.,* **105**(5), 1208–1216.
37. Lippok, S., Radtke, M., Obser, T., Kleemeier, L., Schneppenheim, R., Budde, U., Netz, R. R., and Rädler, J. O. (2016) Shear-induced unfolding and enzymatic cleavage of full-length VWF multimers. *Biophys. J.,* **110**(3), 545–554.
38. Grässle, S., Huck, V., Pappelbaum, K., Gorzelanny, C., Aponte-Santamaría, C., Baldauf, C., Gräter, F., Schneppenheim, R., Obser, T., and Schneider, S. (2014) von Willebrand factor directly interacts with DNA from neutrophil extracellular traps. *Arter. Thromb. Vasc. Biol.,* pp. ATVBAHA–113.
39. Chiu, J. and Chien, S. (2011) Effects of disturbed flow on vascular endothelium: pathophysiological basis and clinical perspectives. *Physiol. Rev.,* **91**(1), 327–387.
40. Schneider, C. A., Rasband, W. S., and Eliceiri, K. W. (2012) NIH Image to ImageJ: 25 years of image analysis. *Nat. Methods,* **9**(7), 671.
41. Aponte-Santamaría, C., Huck, V., Posch, S., Bronowska, A. K., Grässle, S., Brehm, M. A., Obser, T., Schneppenheim, R., Hinterdorfer, P., and Schneider, S. W. (2015) Force-sensitive autoinhibition of the von Willebrand factor is mediated by interdomain interactions. *Biophys. J.,* **108**(9), 2312–2321.
